# Selective GSK3β Inhibition Mediates an Nrf2-Independent Anti-inflammatory Microglial Response

**DOI:** 10.1007/s12035-022-02923-2

**Published:** 2022-06-23

**Authors:** Mohamed H. Yousef, Mohamed Salama, Hassan A. N. El-Fawal, Anwar Abdelnaser

**Affiliations:** 1 School of Sciences and Engineering, Biotechnology Graduate Program, The American University in Cairo, P.O. Box: 74, Cairo, Egypt; 2 Institute of Global Health and Human Ecology, School of Sciences and Engineering, The American University in Cairo, P.O. Box: 74, Cairo, Egypt

**Keywords:** GSK3, Paralog selectivity, Microglia, Neuroinflammation, Neurodegenerative diseases, Oxidative stress, Nrf2, NF-κB

## Abstract

Glycogen synthase kinase 3 (GSK3) is associated with the proinflammatory phenotype of microglia and has been shown to act in concert with nuclear factor kappa B (NF-κB). GSK3 is also a suppressor of nuclear factor erythroid 2-related factor 2 (Nrf2), the principal regulator of redox homeostasis. Agreeing with the oxidative paradigm of aging, Nrf2 is often deregulated in parainflammatory and neurodegenerative diseases. In this study, we aimed to explore a multimodal disease-modifying utility of GSK3 inhibition, beyond neuronal proteopathologies. Furthermore, we aimed to underscore the difference in therapeutic value between the two GSK3 paralogs by isoform-selective chemical inhibition. The anti-inflammatory effects of paralog-selective GSK3 inhibitors were evaluated as a function of the reductive capacity of each to mitigate LPS-induced activation of SIM-A9 microglia. The Griess method was employed to detect the nitrate-lowering capacity of selective GSK3 inhibition. Real-time PCR was used to assess post-treatment expression levels of pro-inflammatory markers and antioxidant genes; pro-inflammatory cytokines were assayed by ELISA. Nuclear lysates of treated cells were examined for Nrf2 and NF-κB accumulation by immunoblotting. Finally, to infer whether the counter-inflammatory activity of GSK3 inhibition was Nrf2-dependent, DsiRNA-mediated knockdown of Nrf2 was attempted. Results from our experiments reveal a superior anti-inflammatory and anti-oxidative efficacy for GSK3β-selective inhibition, compared to GSK3α-selective and non-selective pan-inhibition; hence, use of selective GSK3β inhibitors is likely to be more propitious than non-selective dual inhibitors administered at comparable doses. Moreover, our results suggest that the anti-inflammatory effects of GSK3 inhibition are not Nrf2 dependent.

**Graphical abstract:**

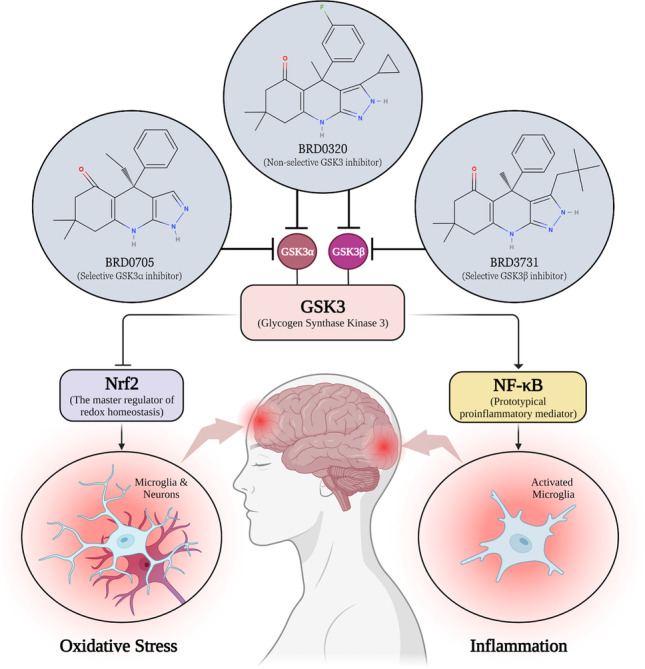

## Introduction and Literature Review

Uncontrolled chronic inflammatory responses are essentially maladaptive and conducive to extensive CNS damage that can go as far as promoting neuronal degeneration [1, 2]. Evidence suggests that neuroinflammation is an integral component of neurodegenerative diseases (NDs) such as Alzheimer’s disease (AD), Parkinson’s disease (PD), and amyotrophic lateral sclerosis (ALS) [3–5]. Presently, microglia constitute the focus of neuroimmunology [6], and in turn, neuroinflammation [7]. Microglia are immunocompetent cells of the monocyte lineage and the resident macrophages of the CNS [8]. If and when an aggravating stimulus is encountered, microglia assume an “activated state,” which is characterized by several phenotypic changes [9]. Dysregulation of microglial activation is a common pathological feature of NDs [10–14].

Within a fairly complex signaling circuitry, glycogen synthase kinase 3 (GSK3), nuclear factor erythroid 2-related factor 2 (Nrf2), and nuclear factor kappa-light-chain-enhancer of activated B cells (NF-κB) constitute a particularly important regulatory loop in neuroinflammation and NDs [15]. Multiple reports suggest signaling cooperativity between the Nrf2/ARE antioxidative pathway and the Wnt/β-catenin pathway, of which GSK3 is a central regulator. The convergence of these two axes was recognized as consequential to glial-mediated inflammation, aging, and neuronal degeneration [16–22]. GSK3 has indeed been proven to serve as a pivotal player within this regulatory network, inhibiting the neuroprotective and antioxidant function of Nrf2 [23–26] and encouraging the inflammatory role of NF-κB [27, 28].

GSK3 is a pleiotropic serine/threonine kinase that has been recognized as the principal tau-phosphorylating kinase, which led to a growing interest in GSK3 as a therapeutic target in neurodegenerative tauopathies [23, 29, 30]. GSK3 was found to mediate the phosphorylation of the majority of abnormally phosphorylated residues in AD [31, 32]. Henceforth, GSK3 has been recognized as a pivotal regulator of toll-like receptor (TLR) signaling, maintaining the balance between proinflammatory and anti-inflammatory responses [33]. GSK3 overactivity incites microglial activation and provokes overproduction of proinflammatory cytokines, chemokines, and nitric oxide (NO) [33–36]. Some studies have gone as far as to conclude that GSK3 gain-of-function is a primary driver in neuroinflammation-mediated neuronal loss [34, 37].

**Fig. 1:**
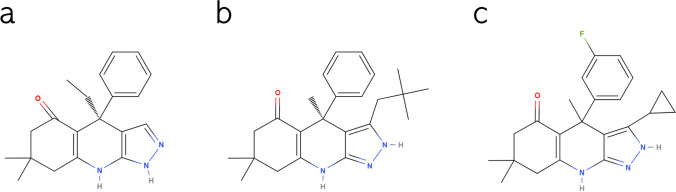
Chemical structure of BRD0705 (GSK3α inhibitor) (**a**), BRD3731 (GSK3β inhibitor) (**b**), and BRD0320 (GSK3α/β non-selective inhibitor) (**c**). Structures were drawn using MolView

Two isoforms (GSK3α and GSK3β) account for GSK3 activity in all mammals [38]. The catalytic domain is conserved between the two GSK3 isoforms with 98% homology [39, 40]. However, within the hinge region, the ATP-binding domain features a switch of the amino acid glutamate (E196) in GSK3α to the amino acid aspartate (D133) in GSK3β, resulting in significant topological and structural disparity between the two paralogs, due to an alteration of hydrogen bonding between the enzyme domains [41]. Unraveling such a subtle difference between GSK3 paralogs, Wagner et al. set out to exploit this topological dissimilitude between the isomers with the purpose of developing paralog-selective GSK3 inhibitors [41]. The team thereupon developed a set of compounds with remarkable selectivity profiles (Fig. 1).

In the current study, we utilize these GSK3 inhibitors to selectively inhibit the GSK3 paralogs in the context of microglial activation. GSK3 is multimodally inculpated in mediating etiopathogenic processes in NDs, both neuronal (proteopathic) and glial (inflammatory). Given the oxidative and inflammatory nature of this disease group in all neural subsets, inhibition of GSK3 can entail a trilateral modulatory utility, whereby its suppression would stunt pathological phosphorylation events associated with injurious misfolded proteins, alleviate glia-mediated neuroinflammatory processes through modulation of NF-kB, and mitigate oxidative damage in both neurons and neuroglia via its regulation of Nrf2. Moreover, given its inability to initiate the pro-malignant β-catenin-driven transcriptional program, the examination of selective inhibition of GSK3α was pursued in comparison with its long-studied paralog.

## Materials and Methods

### Materials

The SIM-A9 murine microglial cell line (CRL-3265™) was purchased from the American Type Culture Collection (ATCC). BRD0705 (GSK3α inhibitor), BRD3731 (GSK3β inhibitor), and BRD0320 (GSK3α/β inhibitor) were kindly provided by the Broad Institute, Inc (Cambridge, MA, USA) in accordance with a material transfer agreement. Sulforaphane (SFN; 10,496) was purchased from Cayman Europe OÜ (Tallinn, Estonia). LPS (Escherichia coli O111:B4; L2630) was purchased from Sigma Chemical Co. (MO, USA). Dulbecco’s modified Eagle medium/Nutrient Mixture F-12 Gibco™ DMEM/F-12, HEPES (31,330,038), Gibco™ Fetal Bovine Serum (10,270,106), Gibco™ Horse Serum, heat inactivated (26,050,070), Gibco™ DPBS, no calcium, no magnesium (14,190,094), dimethyl sulfoxide DMSO (67–68-5), chloroform (HPLC grade; C607SK-1), isopropanol (HPLC grade; BP26324), ethanol (HPLC grade; 64–17-5), RevertAid cDNA kit (K1621), PowerUp™ SYBR™ Green (2X) Master Mix (A25741), mRNA (CD11b, IL-6, iNOS, TNF-α, GAPDH) primers (10,629,186; designed by NCBI primer blast tool), Pierce™ BCA Protein Assay Kit (23,225), and Pierce™ ECL Western Blotting Substrate (32,106) were all acquired from ThermoFisher Scientific (MA, USA). MTT (M6494), Griess Reagent Kit (G7921), NuPAGE™ LDS Sample Buffer (NP0007), NuPAGE™ Reducing Agent (NP0009), NuPAGE™ 10%, Bis–Tris, 1.0 mm, Mini Protein Gels, 10-well (NP0301), 20X NuPAGE™ MES Running Buffer (NP0002), NF-κB p65 polyclonal antibody (PA1-186), and Blocker™ BSA (10%) in PBS (37,525) were obtained from Invitrogen (CA, USA). Penicillin–Streptomycin Mixture Pen/Strep (09-757F) and phosphate-buffered saline (10X) PBS (17-516Q) were supplied by Lonza-Bioscience (Basel, Switzerland). QiAzol lysis buffer (79,306) and nuclease-free water (129,114) were procured from Qiagen (Hilden, Germany). Protease Inhibitor Cocktail (5871), Phosphatase Inhibitor Cocktail (5870), Prestained Protein Marker, Broad Range (11–190 kDa) (13,953), Nrf2 monoclonal antibody (12721 T), β-Catenin (D10A8) XP® Rabbit mAb (8480), and secondary goat anti-rabbit HRP-conjugated antibody (7074P2) were all requisitioned from Cell Signaling (Danvers, MA, USA). 10 × Towbin Buffer (42,558.02), 10 × TBS Buffer (42,596.01), Tween 20 (39,796.01), and Methanol (45,631.02) were from SERVA Electrophoresis GmbH (Heidelberg, Germany). Mouse IL-1β ELISA Kit (E-EL-M0037), Mouse IL-6 ELISA Kit (E-EL-M0044), Mouse TNF-α ELISA Kit (E-EL-M0049), and Lamin B1 Polyclonal Antibody (E-AB-40257) were obtained from Elabscience (Houston, TX, USA). HERAPLUS SYBR® Green qPCR Kit (WF10308001) was purchased from Willowfort (Birmingham, UK) and primers IL-1β, Iba1, c-Myc, HO-1 and Osgin1 (S015950; designed by NCBI primer blast tool) were ordered from Synbio Technologies (Monmouth Junction, NJ, USA). TriFECTa RNAi Kit and PrimeTime Assays for Nrf2 (Mm.PT.58.29108649) and HPRT (Mm.PT.39a.22214828) were supplied by Integrated DNA Technologies (Coralville, IA, USA), and the Minute™ Cytoplasmic and Nuclear Extraction Kit for Cells (SC-003) was bought from Invent Biotechnologies (Plymouth, MN, USA).

### Cell Culture

SIM-A9 cells (CRL-3265™) were maintained in Dulbecco’s modified Eagle medium/Nutrient Mixture F-12 (DMEM/F12) medium supplemented with 10% heat-inactivated fetal bovine serum, 5% heat-inactivated donor horse serum, and 1% Pen-Strep (100 units/mL penicillin, and 100 μg/mL streptomycin) in a 5% CO_2_ humidified incubator at 37 °C.

### Cell Viability: MTT Assay

SIM-A9 cells were seeded in 96-well microtiter plates at a density of 1 × 10^5^ cells/mL and allowed to acclimate overnight. To determine non-cytotoxic concentrations of the experimental compounds, the cells were treated with different concentrations of GSK3 inhibitors (10 μM, 20 μM, 40 μM and 80 μM) for 24 h. Sulforaphane (SFN), used as a positive control for countering inflammation and Nrf2 activation, was also evaluated for its cytotoxicity at different molarities (1 μM, 5 μM, 10 μM). Additionally, varying concentrations (10 ng/mL, 100 ng/mL, and 1 μg/mL) of *E. coli* LPS (O111:B4) were similarly evaluated; toxicity following treatment with 100 ng/mL in conjunction with each GSK3 inhibitor or SFN was also assessed to simulate subsequent experimental conditions. All treatments were prepared in serum-free DMEM/F12 medium. Cell viability following treatments was determined using the MTT colorimetric assay. Following a 24-h incubation of the treatments with the cells, the culture medium was aspirated off and replaced with 100 μL of (1 mg/mL) MTT solution prepared in serum-free DMEM/F12 medium. Subsequent to a further 2-h incubation, the MTT solution was discarded, and the insoluble formazan crystals formed were dissolved in 100 μL DMSO. Absorbance was measured at 540 nm using NanoSPECTROstar microplate reader (BMG LABTECH, Ortenberg, Germany), and cell viability was calculated relative to untreated controls.

### Determination of Nitrite: Griess Method

The concentration of nitrite in the culture medium was determined using the Griess method. The Griess reaction was performed following a treatment protocol in which cells were pretreated with GSK3 inhibitors (10 μM, 20 μM, and 40 μM) or SFN (5 μM) for 2 h and then stimulated with 100 ng/mL LPS for 24 h, with continued exposure to the compounds. Thereafter, 150 μL of cell culture media were diluted with 130 μL of deionized water and 20 μL of the Griess reagent were added to the diluted supernatant. Following a 30-min incubation in the dark, the color of the formed azo chromophore was spectrophotometrically measured at 548 nm using Nano SPECTROstar microplate reader (BMG LABTECH, Ortenberg, Germany). The concentration of nitrite in each sample was computed using the linear equation generated from a standard curve, which was plotted using the recorded absorbance values for various concentrations of a nitrite standard.

### Treatment and Isolation of Total RNA

SIM-A9 cells were seeded in 6-well plates at a density of 1 × 10^6^ cells/mL and allowed to acclimate overnight. The cells were then treated with GSK3 inhibitors (10 μM and 20 μM), or SFN (5 μM) for 2 h. Afterwards, the media were aspirated off and a treatment cocktail preserving the same abovementioned concentrations of the compounds with the addition of LPS at a concentration of 100 ng/mL was added to induce microglial activation in the presence of the GSK3 inhibitors or SFN. Following an additional 6 h incubation, total RNA was extracted by lysing the cells using the phenol/guanidine-based QIAzol Lysis Reagent. First strand cDNA was synthesized from 1 μg of RNA of each sample using the RevertAid First Strand cDNA Synthesis Kit in line with the manufacturer’s guidelines. The mRNA expression levels of inflammation-related genes (CD11b, Iba1, iNOS, IL-1β, IL-6, TNF-α) and Nrf2 target genes (HO-1 and Osgin1) as well as c-Myc (transcriptional target of β-catenin) were quantified by real-time quantitative polymerase chain reaction using the ABI Prism 7500 real-time PCR system (Applied Biosystems, CA, USA). Expression was normalized to GAPDH (or HPRT in post-knockdown qPCR assessment of proinflammatory markers), and relative fold gene expression was computed using the comparative CT (ΔΔCT) method. Specific primer pairs were generated via the NCBI Primer-Blast tool (https://www.ncbi.nlm.nih.gov/tools/primer-blast/) and purchased from ThermoFisher or Synbio Technologies; primer sequences are shown in Table 1.

**Table 1:** List of primers used for qPCR

Target mRNA	Sequence (5′-3′)		Tm (°C)
CD11b	*Forward Primer:*	AAGCAGCTGAATGGAGGAC	55
	*Reverse Primer:*	GGGCCCCATTGGTTTTGTGAA	55
Iba1	*Forward Primer:*	CCTGCAGACTTCATCCTCTC	57
	*Reverse Primer:*	AGGCATCACTTCCACATCAG	55
IL-6	*Forward Primer:*	GATGCTACCAAACTGGATATAATCAG	55
	*Reverse Primer:*	CTCTGAAGGACTCTGGCTTTG	58
IL-1β	*Forward Primer:*	AAAGCTCTCCACCTCAATGG	55
	*Reverse Primer:*	TTGGGATCCACACTCTCCAG	57
iNOS	*Forward Primer:*	GGAACCTACCAGCTCACTCTGG	63
	*Reverse Primer:*	TGCTGAAACATTTCCTGTGCTGT	60
TNF-α	*Forward Primer:*	GAACTCCAGGCGGTGCCTAT	63
	*Reverse Primer:*	TGAGAGGGAGGCCATTTGGG	63
HO-1	*Forward Primer:*	CACAGATGGCGTCACTTCGTC	60
	*Reverse Primer:*	GTGAGGACCCACTGGAGGAG	62
Osgin1	*Forward Primer:*	CGGTGACATCGCCCACTAC	62
	*Reverse Primer:*	GCTCGGACTTAGCCCACTC	62
c-Myc	*Forward Primer:*	AGCTGTTTGAAGGCTGGATT	53
	*Reverse Primer:*	CTGCTGTTGCTGGTGATAGA	55
GAPDH	*Forward Primer:*	CTTTGTCAAGCTCATTTCCTGG	57
	*Reverse Primer:*	TCTTGCTCAGTGTCCTTGC	58

For post-knockdown qPCR experiments, PrimeTime qPCR primers for Nrf2 and HPRT were purchased from Integrated DNA Technologies (Coralville, IA, United States) as predesigned primer assays. The PCR reactions were subjected to the following thermocycling conditions: an initial holding stage run at 95 °C for 10 min, followed by 40 2-step cycles of denaturation at 95 °C for 15 s then annealing/extension for 60 s at 60 °C; data collection was set to occur during step 2. For every gene of interest, ΔCT values were determined for each sample as the difference between the CT values obtained for the target gene and the reporter gene in the same sample, where GAPDH (or HPRT in post-knockdown qPCR assessment of proinflammatory markers) was used as the housekeeping reporter gene. Relative changes in expression of any given gene (ΔΔCT) were then calculated as the difference between the ΔCT of each treatment group and the average ΔCT of the untreated control group. Finally, fold change of expression was determined as 2^−ΔΔCT^, individually calculated for each group.

### Quantification of Secretory Proinflammatory Cytokines Using ELISA

Pre-coated micro-ELISA plates from Elabscience® were used to quantify the protein expression of the proinflammatory cytokines IL-1β, IL-6, and TNF-α in the supernatant of SIM-A9 cells stimulated with LPS in the presence of GSK3 inhibitors or SFN, following the treatment protocol above. In short, the cells were seeded in 6-well plates at a density of 1 × 10^6^ cells/mL. Following overnight incubation, the cells were pretreated with GSK3 inhibitors (20 μM) or SFN (5 μM) for 2 h. The media were then removed and replaced with fresh media containing 100 ng/mL of LPS while maintaining the concentration of GSK3 inhibitors at 20 μM and SFN at 5 μM. The culture media were collected 24 h later, centrifuged at 1000 × g for 20 min at 4 °C and the supernatant was transferred to clean microcentrifuge tubes. For IL-6 and TNF-α, the supernatant was diluted in a ratio of 1:20 in buffered sample diluent, whereas undiluted samples were used for the detection of IL-1β. Optical density was measured at 450 nm using a NanoSPECTROstar microplate reader (BMG LABTECH, Ortenberg, Germany).

### Fractionation of Total Cell Lysates into Nuclear and Cytoplasmic Extracts

In order to differentially evaluate the effect of inhibition of GSK3 paralogs on the transcriptional activity of Nrf2, NF-κB p65, and β-catenin, nuclear lysates of treated SIM-A9 cells were extracted using Minute™ Cytoplasmic and Nuclear Extraction Kit for Cells, according to the manufacturer’s instructions. Briefly, SIM-A9 cells seeded at a density of 1 × 10^6^ cells/mL in 6-well plates were pretreated with GSK3 inhibitors (20 μM) or SFN (5 μM) for 2 h. Following an additional 24 h incubation with 100 ng/mL LPS in the presence of the test compounds, the cells were washed in ice-cold PBS buffer and 300 μL cytoplasmic extraction buffer, containing 1 × Protease Inhibitor Cocktail and 1 × Phosphatase Inhibitor Cocktail, and were added to each well. Cell lysates were transferred to pre-chilled microcentrifuge tubes, vortexed vigorously, and centrifuged at 12,000 × g for 5 min at 4 °C. The supernatant (cytosolic fraction) was transferred to a clean tube and stored at − 80 °C for future use. Nuclear extraction buffer (150 μL), also containing protease and phosphatase inhibitor cocktails, was added to the pelleted nuclei, the mixture forcefully vortexed and incubated on ice for 1 min. The last step was repeated 4 times, alternating between vigorous vortexing and 1 min incubation on ice. The nuclear extracts were then transferred to pre-chilled filter cartridges mounted onto collection tubes and centrifuged at 16,000 × g for 30 s. The filter cartridges were then discarded, and the collected nuclear extracts were stored at − 80 °C until future use.

### Quantification of Cytoplasmic and Nuclear Proteins

Total protein content in the cytoplasmic and nuclear fractions was quantified using the Pierce™ BCA Protein Assay Kit. In a 96-well microtiter plate, 25 μL of each sample and standard dilution were added to the designated wells. Every sample or standard dilution was assayed in triplicates. In each well, 200 μL working BCA reagent were added, the plate was thoroughly mixed on an orbital shaker for 30 s and then incubated at 37 °C for 30 min. The plate was then cooled down, and the absorbance of the formed colored complexes was measured at 562 nm using NanoSPECTROstar microplate reader (BMG LABTECH, Ortenberg, Germany). Sample concentrations were computed from the line equation generated by plotting OD values for varying concentrations of a bovine serum albumin standard.

### Western Blotting

Western blotting was carried out in denaturing and reducing conditions. Samples were prepared in 1 × NuPAGE™ LDS Sample Buffer and 1 × NuPAGE™ Reducing Agent and completed to volume with deionized water. The samples were briefly vortexed, spun down, and heated at 70 °C for 10 min. The samples were then run on NuPAGE™ 10%, Bis–Tris, 1.0 mm, Mini Protein Gels. Gels were run at 200 V for 35 min. For protein transfer, the gels were blotted onto methanol-activated PVDF membranes. Protein transfer was set to run for 1 h at 30 V. Following completion of the transfer, membranes were briefly washed in 1 × TBST buffer and blocked using 5% BSA in 1X TBST for 1 h on an orbital shaker. Following adequate washing, primary anti-mouse Nrf2 monoclonal antibody (1:1000), anti-mouse β-catenin monoclonal antibody (1:1000), anti-mouse NF-κB p65 polyclonal antibody (1:2000), or anti-mouse Lamin B1 polyclonal antibody (1:1000) were added to the membranes and incubated at 4 °C overnight, on a rocking platform. Secondary goat anti-rabbit HRP-conjugated antibody (1:2500) was then added to the membranes and incubated on a shaker for 1 h at room temperature. Pierce™ ECL Western Blotting Substrate was added to the membranes, and chemiluminescence was measured using the ChemiDoc™ Imaging System (Bio-Rad®, CAT# 12,003,153); densitometric analysis of the imaged bands was carried out using the Image Lab 6.1 software, using Lamin B1 as a loading control.

### Transfection of SIM-A9 Cells with Nrf2-Targeting siRNAs

SIM-A9 cells were reverse transfected using 3 different predesigned Nrf2-targeting DsiRNAs, a non-targeting negative control DsiRNA, an HPRT-targeting positive control DsiRNA and a TYE 563 Transfection Control DsiRNA. For complexing, Lipofectamine™ 3000 and the DsiRNAs, both diluted in basal DMEM/F12 medium, were added to each other in a 1:1 ratio, such that the manufacturer-recommended volume of Lipofectamine/well is maintained at 3.75 μL and the formed lipid complex encapsulated 10 nM DsiRNA. The mixture was incubated for 20 min for complete complexing. Afterwards, 750 μL cell suspension (equivalent to 6 × 10^5^ cells/well) and 250 μL of the DsiRNA-lipid complex were added to each well. The plates were thoroughly mixed and incubated in a 5% CO_2_ humidified incubator at 37 °C for 24 h. Fluorescence in cells transfected with the TYE 563-labeled Transfection Control DsiRNA (Absorbance Maximum: 549 nm – Emission Maximum: 563 nm) was checked under Olympus IX70 Inverted Fluorescent Light Microscope at 6 h and 24 h. The next day, the media containing the transfection complex were discarded and the treatment protocol was commenced as hereabove outlined. Following a 6 h incubation with LPS and GSK3 inhibitors (20 μM) or SFN (5 μM), the cells were lysed in QIAzol Lysis Reagent. Total RNA was thereafter extracted and processed for qPCR experiments as previously described.

### Statistical Analysis

One-way analysis of variance (ANOVA) followed by Student–Newman–Keuls post hoc test were applied to determine the statistical significance among the various study groups. A threshold value of 0.5 was set for the probability value (*P-*value), with *P* < 0.05 considered statistically significant. The data are presented as the means ± standard error of the mean (SE) for the designated number of independently executed experiments. Comparative analysis among study groups was carried out via SigmaPlot (Version 14.0; Systat Software, Chicago, IL, USA) and GraphPad Prism (Version 9.0.0; San Diego, CA, USA). Data was graphically rendered in GraphPad Prism.

## Results

### Effect of LPS on the Viability of SIM-A9 Cells

**Fig. 2:**
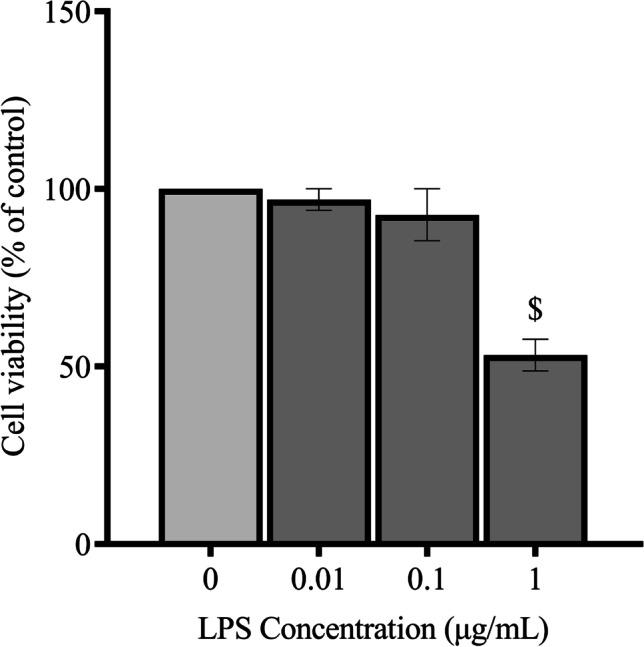
Effect of LPS on viability of SIM-A9 cells. LPS cytotoxicity was assessed using the MTT assay following treatment of SIM-A9 cells (5 × 10.^5^ cells/mL) with 10 ng/mL, 100 ng/mL, and 1 μg/mL LPS. Data is expressed as means ± SEM. Group comparisons were drawn using one-way ANOVA, followed by the Student–Newman–Keuls post hoc test; $*P-*value < 0.05 (relative to negative [untreated] controls)

For the purposes of this study, LPS was used to stimulate a proinflammatory program in SIM-A9 microglia. The MTT assay and the Griess method were conjointly employed to determine the optimal concentration of LPS at which maximal microglial activation can be achieved, without compromising cell viability. To that end, SIM-A9 seeded at varying densities were treated with LPS at the following concentrations: 10 ng/mL, 100 ng/mL, and 1 μg/mL. Subsequent to measuring nitrite levels in the culture media from each group to determine the LPS concentration/cell density combination of the strongest proinflammatory response, the cellular viability of the cells was assayed using the MTT reagent as outlined above. Lower seeding densities were associated with increased cytotoxicity at the 100 ng/mL and the 1 μg/mL concentrations. Cells seeded at a density of 1 × 10^5^ cells/mL fared considerably better (Table 2). On the other hand, seeding densities of 2.5 × 10^5^ cells/mL and 5 × 10^5^ cells/mL only exhibited a significant drop in viability at the 1 μg/mL LPS concentration. However, cells seeded at both these densities maintained acceptable rates of viability for downstream experimentation when treated with 100 ng/mL LPS. LPS administered at 10 ng/mL was not associated with significant cytotoxicity across all cell densities. The percentage change in cell viability following LPS treatment is shown in Table 2. Given that treatment of cells seeded at 5 × 10^5^ cells/mL with LPS at the 100 ng/mL concentration was associated with the highest nitrite levels (as will be shown), while maintaining a good viability profile (Fig. 2), these conditions were adopted for subsequent experiments.

**Table 2:** Effect of LPS on the viability of SIM-A9 cells

Seeding density (cells/mL)	Cell viability for each LPS concentration		
	10 ng/mL	100 ng/mL	1 μg/mL
0.5 × 10^5^	94.97% ± 0.11	66.11% ± 0.26	55.39% ± 0.29
1 × 10^5^	100% ± 0.27	94.28% ± 0.42	81.70% ± 0.19
2.5 × 10^5^	96.96% ± 0.56	92.73% ± 0.4	53.25% ± 0.27
5 × 10^5^	96.50% ± 1.44	93.49% ± 0.99	62.54% ± 2.61

### Effect of BRD0705, BRD3731, BRD0320, and SFN on the Viability of SIM-A9 Cells

**Fig. 3:**
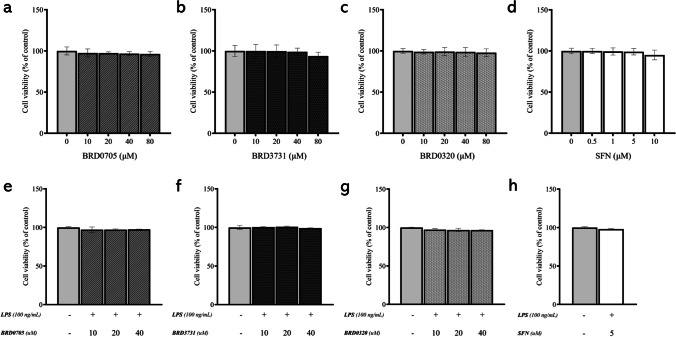
Effect of BRD0705, BRD3731, BRD0320, and SFN on viability of SIM-A9 cells. Cell viability was assessed using the MTT assay following treatment of SIM-A9 cells with BRD0705 (GSK3α inhibitor; **a**), BRD3731 (GSK3β inhibitor; **b**), BRD0320 (GSK3α/β inhibitor; **c**), and SFN (**d**). All experimental compounds were then reassessed in presence of 100 ng/mL LPS (**e**). Data is expressed as means ± SEM. Group comparisons were drawn using one-way ANOVA, followed by the Student–Newman–Keuls post hoc test

The experimental GSK3 inhibitors BRD0705 (GSK3α inhibitor), BRD3731 (GSK3β inhibitor), and BRD0320 (GSK3α/β inhibitor) were evaluated for their cytotoxicity in SIM-A9 cells via the MTT assay, following the hereinabove outlined treatment protocol, which uses 10, 20, 40, and 80 μM concentrations of each compound alone or in conjunction with 100 ng/mL LPS. As evident in Fig. 3, none of the compounds was significantly cytotoxic at any of the tested concentrations, either alone or in the presence of LPS. SFN, used as a positive control for countervailing inflammation and Nrf2 activation, was also assayed for its cytotoxicity at the treatment concentrations of 0.5 μM, 1 μM, 5 μM, and 10 μM. Again, cellular viability was sustained across all treatment concentrations.

### Effect of GSK3 Inhibitors on Nitrite Production in LPS-Stimulated SIM-A9 Cells

**Fig. 4:**
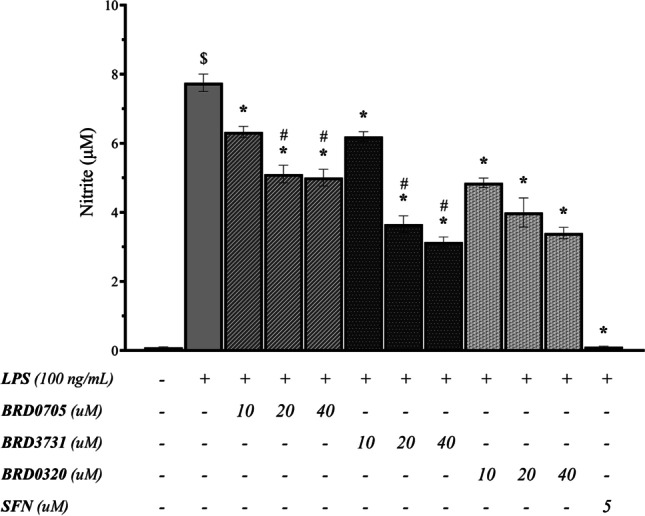
Effect of GSK3 inhibitors on nitrite production in LPS-stimulated SIM-A9 cells. SIM-A9 cell (5 × 10.^5^ cells/mL cell density) was stimulated with 100 ng/mL LPS for 24 h, in the presence of the GSK3 inhibitors and SFN. Nitrite production was measured in the cell culture media using the Griess method. Data is expressed as means ± SEM. Group comparisons were drawn using one-way ANOVA, followed by the Student–Newman–Keuls post hoc test; *P-value < 0.05 (relative to the LPS-stimulated group); $*P-*value < 0.05 (relative to negative [untreated] controls); #*P-*value < 0.05 (BRD0705 at any given concentration relative to BRD3731 at the corresponding concentration)

To initially evaluate any potential anti-inflammatory effect associated with the tested GSK3 inhibitors on a functional level, nitrite production was again measured in the cell culture media of SIM-A9 cells treated with LPS in the presence of the GSK3 inhibitors, according to the protocol previously outlined. In this experiment, LPS significantly increased nitrite levels compared to control (Fig. 4). BRD0705 (GSK3α inhibitor) significantly inhibited the LPS-stimulated production of nitrites by 18.39%, 34.09%, and 35.43% at 10 μM, 20 μM, and 40 μM, respectively. The GSK3β inhibitor, BRD3731, also significantly inhibited LPS-stimulated nitrite production by 20.12%, 52.78%, and 59.44% at 10 μM, 20 μM, and 40 μM, respectively. The anti-nitrosative efficacy of the lowest concentration of the GSK3α/β inhibitor BRD0320 (10 μM) surpassed the highest of BRD0705 (GSK3α inhibitor; 40 μM) as it significantly inhibited the LPS-stimulated production of nitrites by 34.36%. Furthermore, BRD0320 (GSK3α/β inhibitor) significantly inhibited the LPS-stimulated production of nitrites by 48.41% and 56.11% at 20 μM and 40 μM, respectively. Differences between the nitrite-lowering efficacy of the 20 μM and 40 μM concentrations of BRD0705 (GSK3α inhibitor) and BRD3731 (GSK3β inhibitor) were statistically significant, while only at the highest concentration did BRD0705 (GSK3α inhibitor) and BRD0320 (GSK3α/β inhibitor) exhibit any significant differences. Variations between BRD3731 (GSK3β inhibitor) and BRD0320 (GSK3α/β inhibitor) were insignificant across all concentrations. Lastly, SFN significantly inhibited the LPS-stimulated production of nitrites by 98.65% at 5 μM.

### Effect of GSK3 Inhibitors on the mRNA Expression of the Microglial Activation Markers CD11b and Iba1 in LPS-Stimulated SIM-A9 Cells

**Fig. 5:**
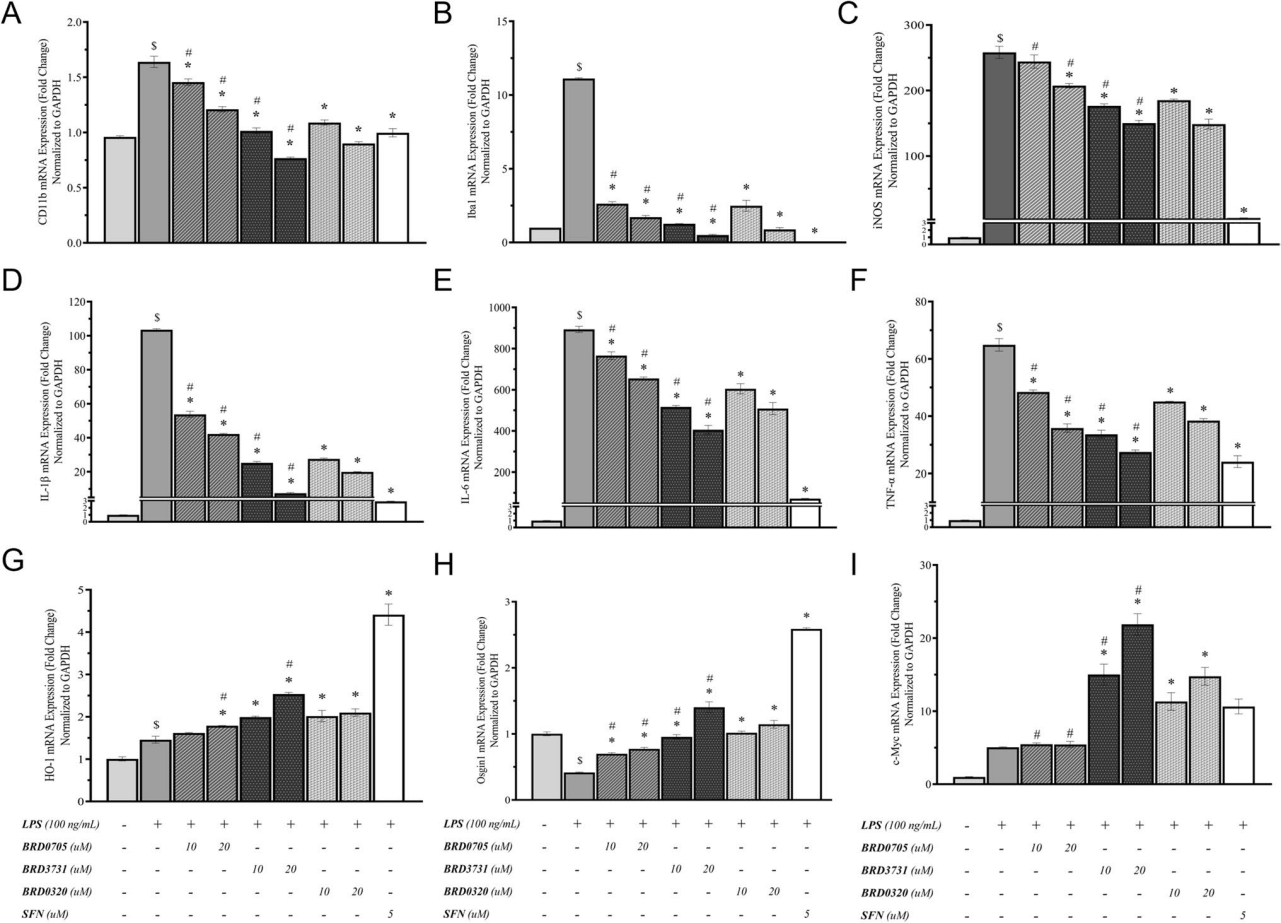
Effect of GSK-3 inhibitors on the mRNA expression of microglial activation, pro-inflammatory markers, Nrf2-driven ARE genes, and β-catenin − transcribed c-Myc in LPS-stimulated SIM-A9 cells. Target mRNA expression was quantified by real-time qPCR and normalized to GAPDH. All compounds show a dose-dependent decrease of the LPS-triggered upregulation of the microglial activation markers CD11b (**a**) and Iba1 (**b**), iNOS (**c**), and the proinflammatory cytokines IL-1β (**d**), IL-6 (**e**), and TNF-α (**f**). All GSK3 inhibitor treatments — except for BRD0705 (GSK3α inhibitor) at 10 μM — show a statistically significant upregulation of the Nrf2-driven ARE gene HO-1 (**a**) and all treatments — without exceptions — correlate with a rise in Osgin1 mRNA levels (**b**). BRD3731 (GSK3β inhibitor) displays the greatest Nrf2-inducing activity after the positive control (SFN, 5 μM), followed by BRD0320 (GSK3α/β inhibitor), then BRD0705 (GSK3α inhibitor). Only BRD3731 (GSK3β inhibitor) and BRD0320 (GSK3α/β inhibitor) show a statistically significant augmentation of c-Myc transcription. BRD3731 (GSK3β inhibitor) displays the most β-catenin − inducing activity followed by BRD0320 (GSK3α/β inhibitor). BRD0705 (GSK3α inhibitor) does not induce any considerable change in c-Myc expression beyond that which is already occasioned by LPS alone. Data is expressed as means ± SEM. Group comparisons were drawn using one-way ANOVA, followed by the Student–Newman–Keuls post hoc test; *P-value < 0.05 (relative to the LPS-stimulated group); $*P-*value < 0.05 (relative to negative [untreated] controls); #*P-*value < 0.05 (BRD0705 at any given concentration relative to BRD3731 at the corresponding concentration)

To further investigate the putative anti-inflammatory effects of selective and non-selective pharmacological GSK3 inhibition, gene expression analysis via quantitative real-time PCR was performed to assess the mRNA levels of principal proinflammatory genes. First, mRNA levels of the commonly assessed surface markers of microglial activation CD11b [34, 42] and Iba1 [43] were quantitatively assessed in treatment-naïve controls in addition to LPS-stimulated cells in the presence and the absence of GSK3 inhibitors. As shown in Fig. 5, stimulation of SIM-A9 microglia by 100 ng/mL LPS resulted in a significant upregulation of CD11b and Iba1. Selective inhibition of GSK3α by 10 μM and 20 μM BRD0705 lowered the mRNA levels of CD11b by 11.22% and 26.2%, and those of Iba1 by 76.37% and 84.53%, respectively. Non-selective inhibition of GSK3 by BRD0320 correlated with an improved reduction of CD11b and Iba1, compared to GSK3α-selective inhibition. CD11b and Iba1 mRNA levels were significantly inhibited by 33.53% and 73.41%, respectively, at 10 μM, and by 45.13% and 80.78%, respectively, at 20 μM BRD0320 (GSK3α/β inhibitor). GSK3β-selective inhibition by BRD3731 was the most potent suppressor of the mRNA expression of these microglial activation markers. As such, CD11b mRNA levels were significantly decreased by 38.11% and 53.23% at 10 μM and 20 μM, respectively. Additionally, Iba1 mRNA levels were significantly inhibited by 88.58% and 95.48% at 10 μM and 20 μM, respectively. The dose-dependent amelioration of function was statistically significant for all three compounds, and at 20 μM, the functional differences between all treatments were statistically meaningful, with the exception of Iba1 modulation by BRD3731 (GSK3β inhibitor) and BRD0320 (GSK3α/β inhibitor). Finally, SFN at 5 μM significantly inhibited CD11b and Iba1 mRNA levels by 43.11% and 99.72%, respectively.

### Effect of GSK3 Inhibitors on the mRNA Expression of iNOS in LPS-Stimulated SIM-A9 Cells

In verification of results obtained from the Griess assay, iNOS, a major player in the inflammatory and para-inflammatory disorders [44, 45] was evaluated for its transcriptional rates in the different treatment groups versus untreated controls. The expression of iNOS significantly increased in LPS-stimulated microglia (Fig. 5). BRD0705 (GSK3α inhibitor) insignificantly inhibited LPS-stimulated iNOS mRNA levels by 4.82% at 10 μM, and it significantly inhibited iNOS mRNA levels by 28.8% at 20 μM. Dual inhibition of the two GSK3 isoforms by BRD0320 resulted in significant inhibition of LPS-stimulated iNOS mRNA levels by 23.99% at 10 μM and 31.4% at 20 μM, whereas GSK3β-selective inhibition by BRD3731 significantly inhibited iNOS mRNA levels by 26.4% at 10 μM and 34.38% at 20 μM. As evident in Fig. 7, SFN at 5 μM significantly inhibited iNOS mRNA levels by 97.77%.

### Effect of GSK3 Inhibitors on the mRNA Expression Levels of the Proinflammatory Cytokines IL-1β, IL-6, and TNF-α in LPS-Stimulated SIM-A9 Cells

Stimulated proinflammatory cytokine signaling is typical of activated microglia and is associated with neuroinflammation and neurodegenerative proteopathy [46]. mRNA expression of the secretory proinflammatory cytokines IL-1β, IL-6, and TNF-α was therefore performed and echoed the results obtained for CD11b, Iba1, and iNOS. LPS significantly increased IL-1β, IL-6, and TNF-α mRNA levels. BRD0705 (GSK3α inhibitor) significantly inhibited IL-1β mRNA levels by 48.09% and 59.22% at 10 μM and 20 μM, respectively. Importantly, BRD3731 (GSK3β inhibitor) significantly inhibited IL-1β mRNA levels by 75.67% and 92.75% at 10 μM and 20 μM, respectively. Additionally, BRD0320 (GSK3α/β inhibitor) significantly inhibited IL-1β mRNA levels by 73.41% and 80.78% at 10 μM and 20 μM, respectively. SFN significantly inhibited IL-1β mRNA levels by 97.17% at 5 μM. The same trend was observed for IL-6; BRD0705 (GSK3α inhibitor) significantly inhibited IL-6 mRNA levels by 14.26% and 26.69% at 10 μM and 20 μM, respectively. BRD3731 (GSK3β inhibitor) significantly inhibited IL-6 mRNA levels by 42.14% and 54.57% at 10 μM and 20 μM, respectively. BRD0320 (GSK3α/β inhibitor) significantly inhibited IL-6 mRNA levels by 32.29% and 43.06% at 10 μM and 20 μM, respectively, whereas SFN significantly inhibited IL-6 mRNA levels by 91.92% at 5 μM.

Post-treatment TNF-α transcriptional modulation followed a similar course. TNF-α levels dropped by 40.8% and 62.87% following BRD3731 (GSK3β inhibitor) treatment at 10 μM and 20 μM, respectively. BRD0320 (GSK3α/β inhibitor) lowered LPS-exacerbated TNF-α levels by 34.55% at 10 μM and by 52.66% at 20 μM, while BRD0705 (GSK3α inhibitor) brought on a 17.94% decline in TNF-α transcripts at 10 μM, almost doubling to 37.6% at 20 μM. A 70.51% reduction in TNF-α levels was registered for SFN (5 μM). The evident dose-dependency was statistically powerful as relayed by the analysis of data retrieved for the 10 μM and 20 μM concentrations. The functional differences between BRD0705 (GSK3α inhibitor) and BRD3731 (GSK3β inhibitor) were also significant, from a statistical standpoint. Only for TNF-α, the modulatory variance between BRD0705 (GSK3α inhibitor) and BRD0320 (GSK3α/β inhibitor) was, however, not statistically significant (Fig. 5).

### Effect of GSK3 Inhibitors on the mRNA Expression of Nrf2-Driven ARE Genes HO-1 and Osgin1 in LPS-Stimulated SIM-A9 Cells

In order to evaluate the effects of selective suppression of the individual GSK3 paralogs on mediating Nrf2 transcriptional activation, the mRNA expression of two ARE genes, HO-1 and Osgin1, was quantified by real-time PCR, following treatment with the GSK3 inhibitors, under the supposition that Nrf2 destabilization and nuclear localization should translate to enhanced transcription of its target genes. The results are shown in Fig. 5. BRD3731 (GSK3β inhibitor) was the most potent Nrf2 activator; it induced a 36.56% enhancement of HO-1 expression at 10 μM and 73.99% at 20 μM, compared to untreated LPS-stimulated microglia. Osgin1 mRNA expression was similarly enhanced by 125.69% at 10 μM BRD3731 (GSK3β inhibitor) and by 237.19% at 20 μM. BRD0320 (GSK3 α/β inhibitor) increased HO-1 mRNA expression by 38.21% and 43.86% at 10 μM and 20 μM, respectively, whereas these concentrations brought about a 144.52% and a 175.08% increase in Osgin1 mRNA levels. BRD0705 (GSK3α inhibitor) at 10 μM treatment stimulated HO-1 transcription by 10.89% and by 22.66% at 20 μM. Osgin1 expression was upraised by 58.56% at the 10 μM concentration and by 67.87% at the 20 μM concentration. With BRD0705 (GSK3α inhibitor), dose variations were of no statistical import for either target, and neither were the differences in functional competency with BRD0320 (GSK3α/β inhibitor) for HO-1.

### Effect of GSK3 Inhibitors on the mRNA Expression of the β-Catenin − Transcribed c-Myc in LPS-Stimulated SIM-A9 Cells

Given empirical evidence suggesting isoform-distinctive β-catenin regulatory profiles [41] and other data herein cited (see Discussion) that pinpoints the involvement of β-catenin in our pathophysiological context of interest, we set out to evaluate c-Myc mRNA levels, a transcriptional target of Wnt/β-catenin signaling pathway [47]. LPS alone incited a 404.79% rise in c-Myc levels. BRD0705 (GSK3α inhibitor) elaborated on this increase by 8.29% (446.61% overall increase relative to untreated controls) at 10 μM and by 12.81% (469.46% overall increase relative to untreated controls) at 20 μM. Selective inhibition of GSK3β by BRD3731 displayed the most β-catenin destabilization, as shown by the elevated c-Myc mRNA levels; BRD3731 (GSK3β inhibitor) upregulated c-Myc by 1399.93% at 10 μM and by 2086.86% at 20 μM, relative to untreated controls. This trend translates to a 197.14% and a 333.22% increase in c-Myc expression at 10 and 20 μM, respectively, compared to cells solely treated with LPS. Non-selective GSK3 inhibition by BRD0320 promoted c-Myc transcription by 1031.67% at 10 μM and 1376.07% at 20 μM, compared to untreated controls (124.19% and 192.41% relative to LPS-stimulated cells). Coupling the insignificance of dose-dependent biological outcome, no statistical significance was demonstrable between the two concentrations of BRD0705 (GSK3α inhibitor). Nonetheless, variations in the pattern of c-Myc mRNA expression were significant statistically across all three compounds, wherever comparisons between matched concentrations were made. Results are shown in Fig. 5.

### Effect of GSK3 Inhibitors on the Protein Levels of the Proinflammatory Cytokines IL-1β, IL-6, and TNF-α in LPS-Stimulated SIM-A9 Cells

**Fig. 6:**
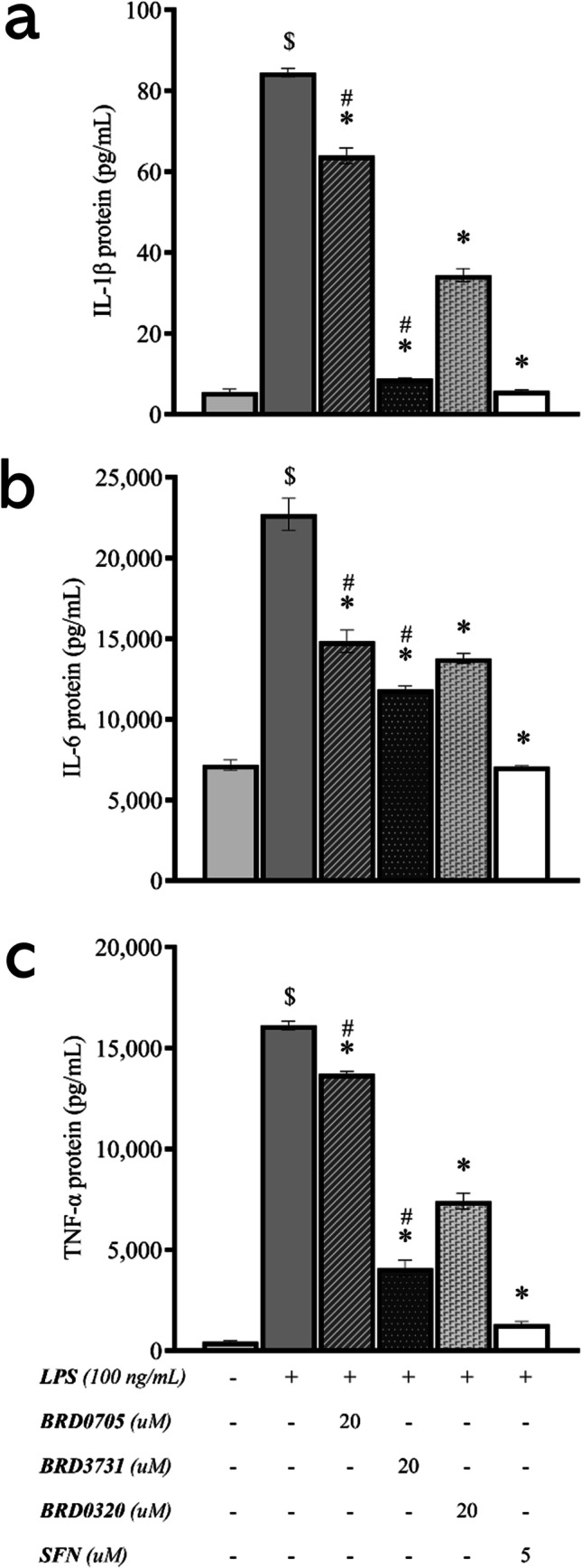
Effect of GSK3 inhibitors on the protein levels of the proinflammatory cytokines IL-1β, IL-6, and TNF-α in LPS-stimulated SIM-A9 cells. All compounds show a statistically significant reduction of the LPS-stimulated upregulation of the proinflammatory cytokines IL-1β (**a**), IL-6 (**b**), and TNF-α (**c**). Data is expressed as means ± SEM. Group comparisons were drawn using one-way ANOVA, followed by the Student–Newman–Keuls post hoc test; *P-value < 0.05 (relative to the LPS-stimulated group); $*P-*value < 0.05 (relative to negative [untreated] controls); #*P-*value < 0.05 (BRD0705 at any given concentration relative to BRD3731 at the corresponding concentration)

To confirm that the anti-inflammatory responses observed on the mRNA level carried through to the protein level, the protein concentrations of the secretory proinflammatory cytokines IL-1β, IL-6, and TNF-α were evaluated by ELISA in the cell culture supernatant. LPS (100 ng/mL) promoted the proinflammatory profile of SIM-A9 cells as reflected by the elevated protein levels of the evaluated cytokines (Fig. 6). At 20 μM, BRD0705 (GSK3α inhibitor) reduced the LPS-mediated increase of IL-1β by 24.33%, IL-6 by 34.67%, and TNF-α by 14.9%. At the same concentration, BRD3731 (GSK3β inhibitor) reduced IL-1β by 89.57%, IL-6 by 47.85%, and TNF-α by 74.62%. Dual inhibition of the two GSK3 isoforms by BRD0320 at 20 μM corresponded with 59.27%, 39.33%, and 54.01% reduction in LPS-elevated IL-1β, IL-6, and TNF-α proteins, respectively. Statistical analysis inferred the significance of all changes noted between the compounds, excepting the instance of comparing BRD0705 (GSK3α inhibitor) and BRD0320 (GSK3α/β inhibitor) within the IL-6 assay.

### Effect of GSK3 Inhibitors on the Nuclear Translocation of Nrf2, β-Catenin, and the p65 Subunit of NF-κB in LPS-Stimulated SIM-A9 Cells

**Fig. 7:**
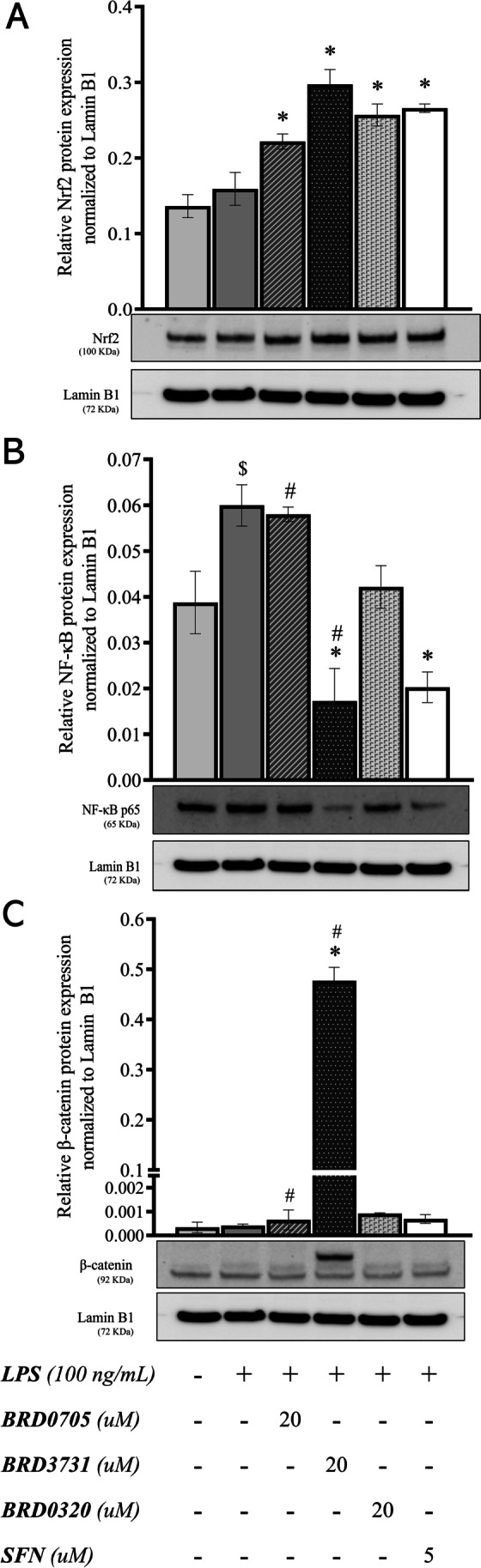
Effect of GSK3 inhibitors on the nuclear translocation of Nrf2, β-catenin, and the p65 subunit of NF-κB in LPS-stimulated SIM-A9 cells. Western immunoblots and derived graphical representation of the protein expression pattern for Nrf2 (**a**), NF-κB p65 (**b**), and β-catenin (**c**) are shown. Data is expressed as means ± SEM. Group comparisons were drawn using one-way ANOVA, followed by the Student–Newman–Keuls post hoc test; *P-value < 0.05 (relative to the LPS-stimulated group); $*P-*value < 0.05 (relative to negative [untreated] controls);; #*P-*value < 0.05 (BRD0705 at any given concentration relative to BRD3731 at the corresponding concentration)

To probe into the mechanistic underpinnings of the observed anti-inflammatory and anti-oxidative effects of GSK3 inhibition, nuclear protein lysates extracted from treated SIM-A9 cells were analyzed by immunoblotting for a quantitative assessment of the levels of Nrf2 and NF-κB p65 in the nucleus. The experiment was to inform whether the modulatory effects of GSK3 inhibition occur via facilitating the translocation of Nrf2 and the p65 subunit of NK-κB into the nuclear compartment, thereby promoting the transcription of their downstream targets. Figure 7 shows images of the immunoblots and the derived graphical representations outlining the treatment-associated modulatory trends. Nrf2 immunoblots revealed an 86.83% increase in nuclear Nrf2 in LPS-stimulated cells concurrently treated with the GSK3β inhibitor, BRD3731 (118.04% increase relative to untreated controls). LPS-stimulated cells treated with the non-selective GSK3 inhibitor, BRD0320, demonstrated a 61.4% increase in nuclear Nrf2 (88.36% increase relative to untreated controls), while BRD0705 (GSK3α inhibitor) raised the nuclear content of Nrf2 by 39.34% compared to cells treated with LPS only (62.62% more than the nuclear Nrf2 levels of untreated controls). SFN, an established electrophilic activator of Nrf2, brought about a 67.06% increase in nuclear Nrf2 compared to LPS-stimulated cells and a 94.97% increase compared to untreated controls. NF-κB immunoblots displayed a contrary trend, where GSK3 inhibition correlated with diminished nuclear levels of NF-κB p65. LPS-stimulated SIM-A9 microglia accumulated more NF-κB p65 in their nuclei (64.17% increase); SFN lowered this augmentation by 74.44%. Again, GSK3β inhibition by BRD3731 exhibited an unmatched anti-inflammatory tendency with an 82.69% drop in nuclear NF-κB p65. Pan-inhibition of GSK3 by BRD0320 in LPS-stimulated cells correlated with a 35.32% decrease in nuclear NF-κB p65. BRD0705 (GSK3α inhibitor) had almost no effect on NF-κB p65 nuclear translocation, with only a 4.01% reduction in the nuclear content of the p65 subunit. Unlike the discrepancy above outlined for Nrf2, the functional unevenness displayed by the densitometric analysis was substantiated by the statistical analysis of the data, where differences between BRD0705 (GSK3α inhibitor) and BRD3731 (GSK3β inhibitor) were statistically meaningful, as well as BRD3731 (GSK3β inhibitor) and BRD0320 (GSK3α/β inhibitor). Wherever comparisons were drawn between BRD0705 (GSK3α inhibitor) and BRD0320 (GSK3α/β inhibitor), differences therewith were determined to be statistically inconsequential. The same statistical outcome was replicated in the analysis of data from β-catenin blots, where BRD3731 (GSK3β inhibitor) was the only GSK3 inhibitor to mediate a significant increase of β-catenin in the nucleus.

### Efficiency of DsiRNA-Mediated Transfection of SIM-A9 Cells

**Fig. 8:**
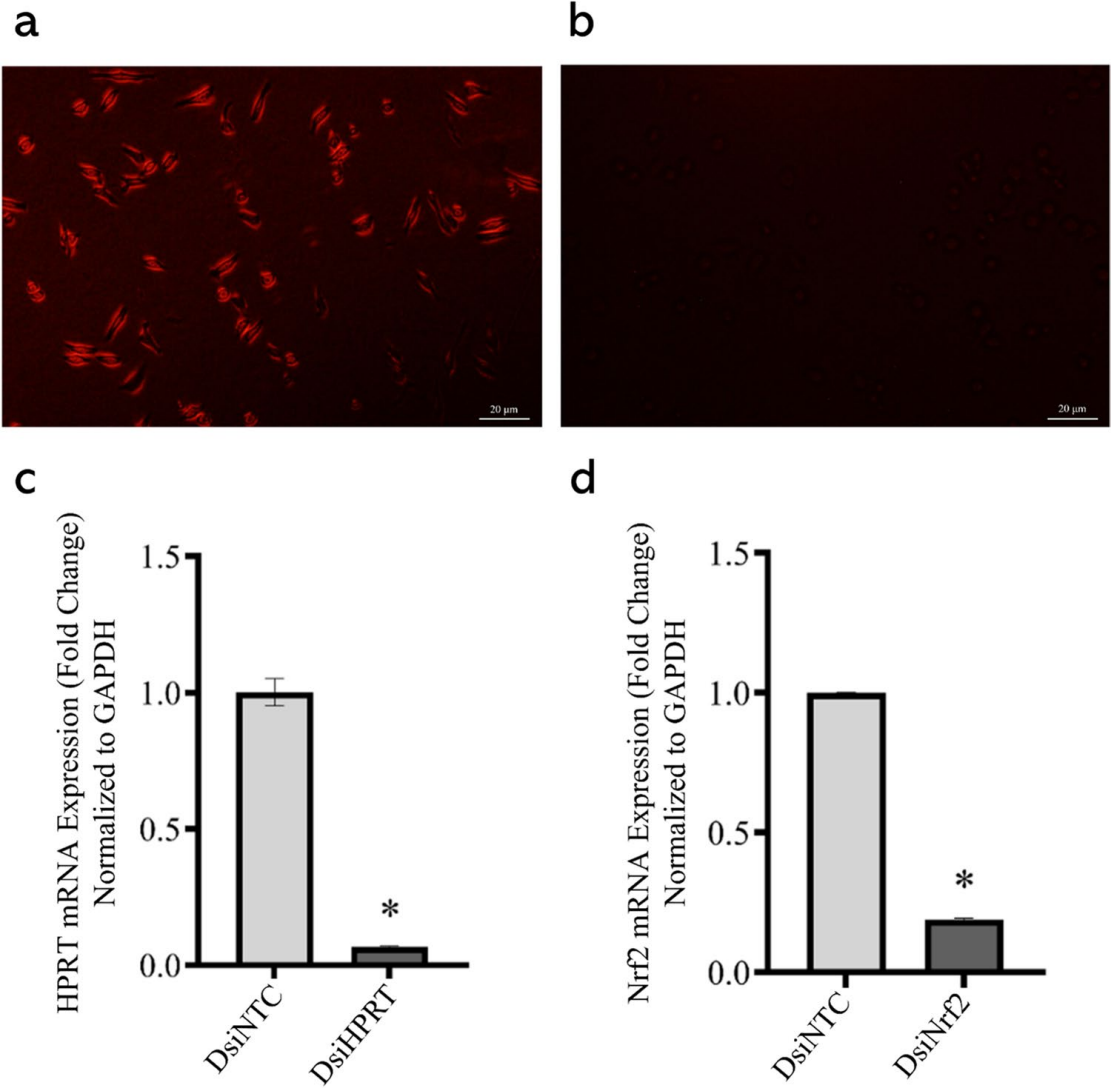
Efficiency of DsiRNA-mediated transfection of SIM-A9 cells. Qualitative evaluation of the transfection protocol shows fluorescence of cells transfected with TYE 563-tagged DsiRNA (**a**) as opposed to untransfected cells (**b**), which do not manifest any discernable fluorescence. Efficiency of transfection was gauged by quantitative measurement of HPRT mRNA expression, following transduction of cells with the positive control HPRT-targeting DsiRNA (**c**). Silencing of Nrf2 was similarly confirmed by qPCR (**d**), following transfection of Nrf2-targeting DsiRNAs into cells. Data is expressed as means ± SEM. Group comparisons were drawn using Welch’s *t*-test; *P-value < 0.05 (relative to the NTC group)

**Fig. 9:**
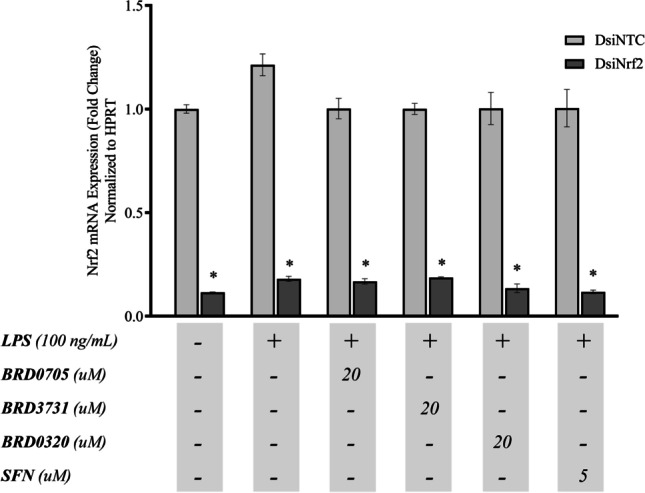
Knockdown efficiency of Nrf2 in DsiNrf2-transfected SIM-A9 cells. Real-time qPCR data showing the expression of Nrf2 in DsiNrf2-transfected SIM-A9 cells relative to their DsiNTC-transfected controls. Nrf2 knockdown efficiency in the DsiNrf2-transfected cells is expressed as the % decrease of Nrf2 expression in the corresponding siNTC-transfected cells. Data is expressed as means ± SEM. Comparisons between each DsiNTC and its respective DsiNrf2 were drawn using Welch's t-test; * *P-*value < 0.05 (relative to the DsiNTC group)

To determine whether the anti-inflammatory effect of the GSK-3 inhibitors was Nrf2-dependent, DsiRNA-mediated knockdown of Nrf2 was pursued as described in the “Materials and Methods” section. Robustness of the transfection protocol was ascertained in two ways; first, SIM-A9 cells were reverse-transfected with a transfection control DsiRNA, tagged with TYE 563. As evident in Fig. 8 a and b, only transfected cells showed fluorescence as soon as 6 h post-transfection, in comparison with non-transfected control cells. For a quantitative assessment of transfection efficiency, an HPRT-targeting positive control DsiRNA was transduced into the cells; HPRT mRNA expression was thereafter assessed, 24 h post-transfection. In cells transfected with the positive control DsiRNA, HPRT was silenced by 93.33% compared to cells transfected with a non-targeting negative control DsiRNA (Fig. 8c). Following the same protocol, in two separate experiments, successful knockdown of Nrf2 was achieved in GSK3 inhibitor-naïve cells, as indicated by post-transfection qPCR, with an efficiency of 87.26% (Fig. 8d) and 88.47% (Fig. 9) compared to cells treated with a non-targeting negative control DsiRNA. Knockdown efficiency across all treatment groups was subsequently determined and ranged between 81.39% to 88.47% (Fig. 9).

### Effect of GSK3 Inhibitors on the mRNA Expression of Nrf2-Driven ARE Genes HO-1 and Osgin1 in DsiNrf2-Transfected, LPS-Stimulated SIM-A9 Cells

**Fig. 10:**
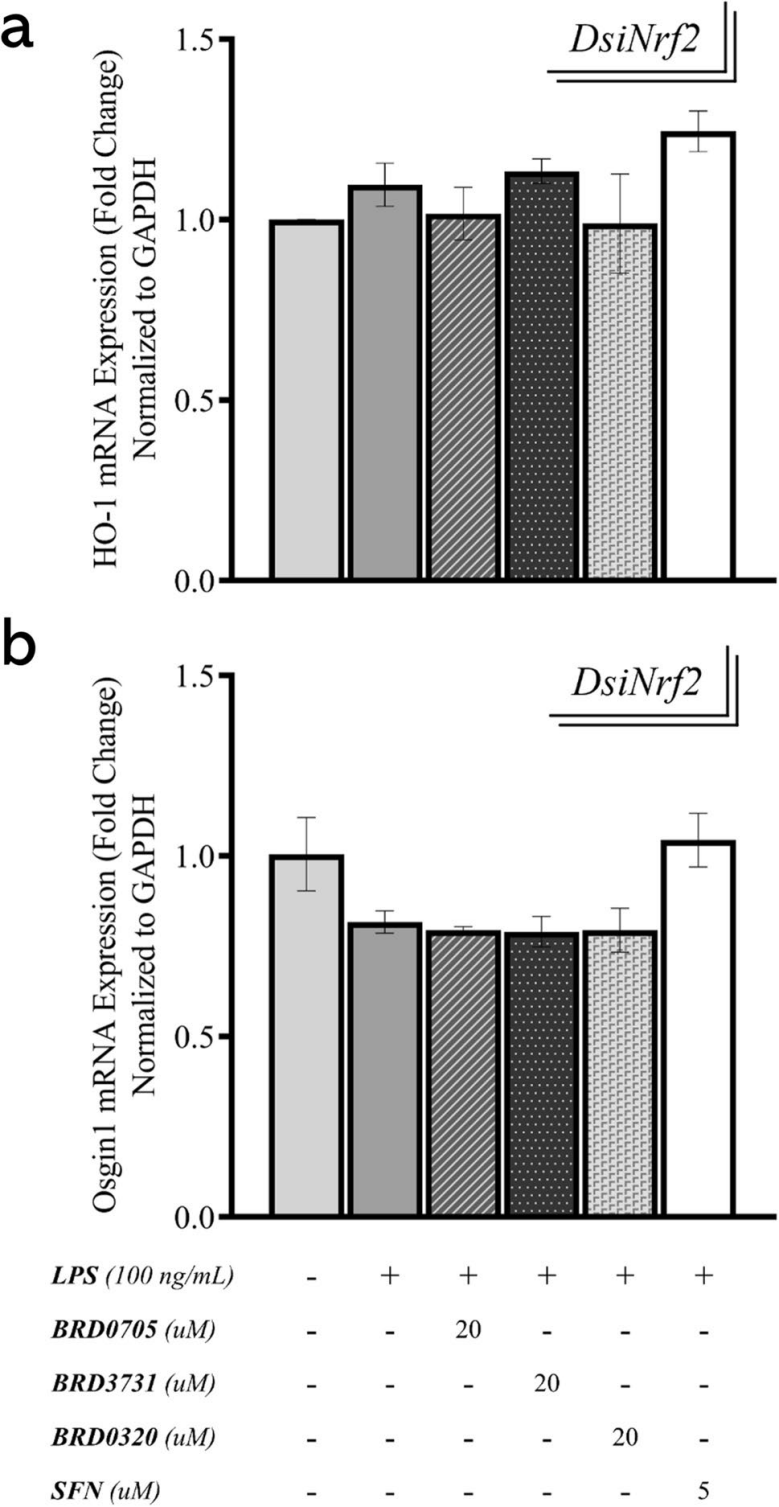
Effect of GSK3 inhibitors on the mRNA expression of Nrf2-driven ARE genes HO-1 and Osgin1 in DsiNrf2-transfected, LPS-stimulated SIM-A9 cells. Both HO-1 (**a**) and Osgin1 (**b**) only exhibit basal levels of expression post-knockdown of Nrf2 and are not affected by any of the treatments. Data is expressed as means ± SEM. Group comparisons were drawn using one-way ANOVA, followed by the Student–Newman–Keuls post hoc test, and relay no intergroup statistical significance (*P-*value > 0.05)

As explained above, Nrf2 mRNA expression was determined to considerably drop, following DsiNrf2-mediated gene silencing. To confirm a corresponding transcriptional restriction of ARE genes, HO-1 and Osgin1 were re-assessed by qPCR, following the knockdown of their transcriptional activator. The generated qPCR data suggests that there was no significant difference in HO-1 and Osgin1 mRNA expression between untreated controls, LPS-stimulated, and LPS/GSK-3 inhibitor-treated groups (Fig. 10).

As with previous qPCR experiments, the Nrf2-activating capability of the GSK3 inhibitors was assessed in the proinflammatory (LPS-stimulated) context; i.e., expression in the GSK3 inhibitor-treated groups was compared to the LPS-stimulated group, rather than the untreated controls. In the cells in which Nrf2 was silenced (DsiNrf2 group), BRD0705 (GSK3α inhibitor)-mediated induction of HO-1 and Osgin1 was reduced by 34.4% and 18.94%, respectively, relative to groups receiving a non-targeting negative control DsiRNA (DsiNTC group). The Nrf2-inducing activity of BRD3731 (GSK3β inhibitor) noted in the DsiNTC group (122.73% increase in HO-1 and 152.42% increase in Osgin1) was likewise compromised by 119.51% for HO-1 and by 151% for Osgin1 in conditions of Nrf2 functional insufficiency (DsiNrf2 group). BRD0320 (GSK3α/β inhibitor) also lost its Nrf2-activating functionality as evidenced by a 66.37% and a 69.81% drop in HO-1 and Osgin1 mRNA expression, respectively. SFN, a well-recognized Nrf2 activator, lost its ability to induce these Nrf2 targets by 195.12% (HO-1) and 348.82% (Osgin1), a substantial reversal of effect, given its remarkable efficacy in stimulating the transcription of these targets in the DsiNTC group (198.1% and 350.3% upregulation of HO-1 and Osgin1, respectively). Collectively, a loss of the anti-oxidative upregulation of HO-1 and Osgin1 by the GSK3 inhibitors was noted in Nrf2 knockdowns (DsiNrf2) compared to controls (DsiNTC).

### Effect of GSK3 Inhibitors on the mRNA Expression of iNOS in DsiNrf2-Transfected, LPS-Stimulated SIM-A9 Cells

**Fig. 11:**
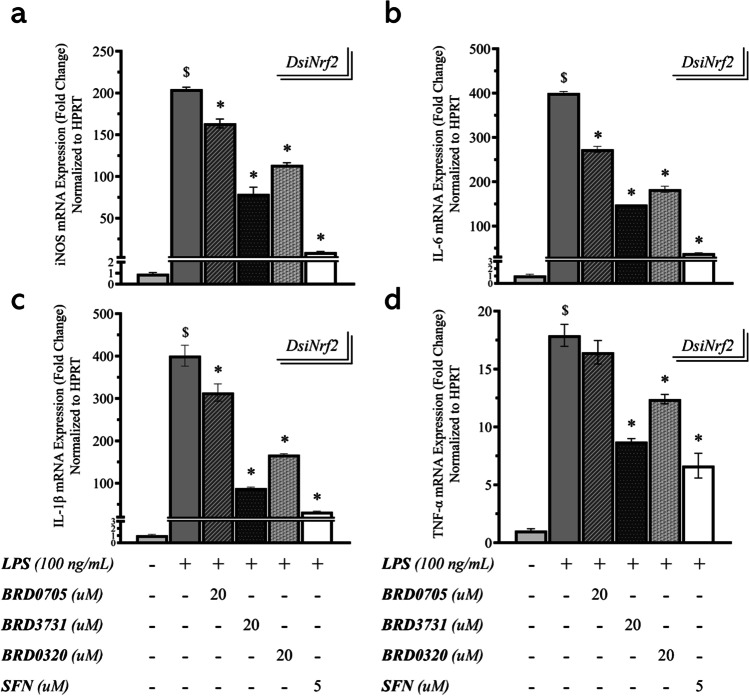
Effect of GSK3 inhibitors on the mRNA expression of the proinflammatory mediators iNOS, IL-1β, IL-6, and TNF-α in DsiNrf2-transfected, LPS-stimulated SIM-A9 cells. Target mRNA expression was quantified by real-time qPCR and normalized to HPRT. GSK3 inhibitors are shown to maintain the same pre-knockdown modulatory pattern of iNOS (**a**), IL-1β (**b**), IL-6 (**c**), and TNF-α (**d**) expression, following Nrf2 knockdown. No significance, biological or statistical, was observed between the modulatory trends effected by GSK3 inhibitors in DsiNrf2 and DsiNTC. Data is expressed as means ± SEM. Group comparisons were drawn using one-way ANOVA, followed by the Student–Newman–Keuls post hoc test; *P-value < 0.05 (relative to the LPS-stimulated group); $*P-*value < 0.05 (relative to negative [untreated] controls); #*P-*value < 0.05 (BRD0705 at any given concentration relative to BRD3731 at the corresponding concentration)

Having confirmed post-knockdown transcriptional suppression of Nrf2-driven genes, transcripts for proinflammatory genes were quantified by qPCR in the DsiNrf2 groups to determine whether Nrf2 silencing would compromise the above-proven anti-inflammatory function of the GSK3 inhibitors. Overall, previously relayed expression patterns remained unchanged. Reduction of iNOS mRNA expression in the DsiNrf2 group amounting to only 3.26% (BRD0705; GSK3α inhibitor), 2.34% (BRD3731; GSK3β inhibitor), 1.68% (BRD0320; GSK3α/β inhibitor), and 2.64% (SFN) of corresponding expression trends in the DsiNTC group suggested no overlap between GSK3-mediated Nrf2 activation and its repressive influence on iNOS transcription (Fig. 11). Comparisons of treatment groups in which Nrf2 was silenced (DsiNrf2) and those in which Nrf2 expression was maintained at its basal levels (DsiNTC) determined lack of any statistical significance of the observed intergroup changes, while analysis of variations across treatments confirmed the soundness of thus far proven BRD3731 (GSK3β inhibitor) superiority to BRD0705 (GSK3α inhibitor) and BRD0320 (GSK3α/β inhibitor).

### Effect of GSK3 Inhibitors on the mRNA Expression of the Proinflammatory Cytokines IL-1β, IL-6, and TNF-α in DsiNrf2-Transfected, LPS-Stimulated SIM-A9 Cells

Conforming to the preceding modulatory tendency, transcription rates for IL-1β, IL-6, and TNF-α were largely analogous between the DsiNrf2 and DsiNTC groups (Fig. 11). This was demonstrable by both the statistical and biological insignificance of the expression pattern differences between both groups. The GSK3 inhibitors comparably reversed LPS-augmented mRNA levels of the screened proinflammatory cytokines. IL-1β mRNA expression in the DsiNrf2 group only decreased by 1.68% (BRD0705; GSK3α inhibitor), 2.71% (BRD3731; GSK3β inhibitor), 2.71% (BRD0320; GSK3α/β inhibitor), and 0.04% (SFN), compared to the DsiNTC group. In relation to the DsiNTC group, levels of IL-6 mRNA in the DsiNrf2 group merely differed by 2.94% (BRD0705; GSK3α inhibitor), 2.65% (BRD3731; GSK3β inhibitor), 7.7% (BRD0320; GSK3α/β inhibitor), and 3.59% (SFN). The DsiNTC group came ahead of the DsiNrf2 group with only 1.15% (BRD0705; GSK3α inhibitor), 4.87% (BRD3731; GSK3β inhibitor), 2.03% (BRD0320), and 3.18% (SFN) higher TNF-α mRNA expression. Collectively and unlike observations made with the anti-oxidative genes HO-1 and Osgin1, the anti-inflammatory effects of GSK3 inhibition were unaffected by Nrf2 silencing, as conveyed by the consistent expression patterns of proinflammatory markers between the DsiNrf2 and DsiNTC groups. Comparisons drawn between BRD0705 (GSK3α inhibitor), BRD3731 (GSK3β inhibitor), and BRD0320 (GSK3α/β inhibitor) once again informed of the statistical import of the differences detected therewith.

## Discussion

Chronically activated and oxidatively stressed microglia are major aggravators of degenerative neuronal damage and their frequently exaggerated proinflammatory response often results in further disease dissemination leading to worsened glial dysfunction and neuronal detrition. While GSK3 has long been recognized as the principal kinase implicated in tau hyperphosphorylation as well as amyloidopathy in neurons, it has more recently been proven to encourage the frenetic proinflammatory alias of microglia. This is particularly germane within chronically stressed microglia. Given that GSK3 is one of the key negative regulators of Nrf2, the redox master of the cell, the regulatory loop therein poses great relevance to the involvement of GSK3 in NDs, not just through its pro-proteopathic influence, but by way of promoting oxidative changes in microglial cells. Moreover, the molecular cooperativity between GSK3 and NF-κB, a major inflammatory player, and the inverse relationship between the latter and Nrf2 suggests an oxidative, proinflammatory GSK3/NF-κB/Nrf2 regulatory loop.

As such, we theorized that inhibitory targeting of GSK3 could pose a multimodal therapeutic strategy in NDs, since not only will it restrict the production of toxic protein forms, but also, it should activate counter-oxidative mechanisms and repress subcellular inflammatory processes in neurons and neuroglia. It was under this conception that we sought to explore the effects of GSK3 inhibition in activated microglia in relation to NF-κB and Nrf2. Moreover, driven by the dearth of research endeavors characterizing the functional differences between GSK3 isoforms, especially in relation to microglial Nrf2 activation and NF-κB-driven inflammation, we aimed to explore the differences between selective inhibition of these isoforms in terms of anti-oxidative and anti-inflammatory potential.

We chose a simple but reliable in vitro model for a reductionistic examination of the differences between such paralog-selective inhibition. Unlike microglial cell lines generated via viral transduction, such as BV2 and N9, SIM-A9 cells are spontaneously immortalized microglia derived from murine cerebral cortices [48]. SIM-A9 cells were found to retain microglial characteristics for as long as 40 passages [49], without genetic or pharmacological manipulation [48]. As such, SIM-A9 cells are expected to closely resemble primary microglia than their virally or pharmacologically transformed counterparts [48]. LPS has been previously used to activate microglia and promote p-tau in animal models of AD [50] and was therefore used as an alternative to recombinant cytotoxic tau oligomers to stimulate activation of SIM-A9 cells in this study, while still maintaining pertinence to the pathological context in question. A patented GSK3 inhibitor kit developed by a team at Broad Institute Inc. [41] was used for paralog-selective inhibition of GSK3, where BRD0705 was used as a GSK3α-selective inhibitor, BRD3731 served as a GSK3β-selective inhibitor and BRD0320 employed for non-selective inhibition of GSK3. As the traditional go-to positive control for Nrf2 activation, SFN was chosen for this purpose in this study.

First, we examined the nitrite-lowering effect of GSK3 inhibitors following microglial activation by LPS as a preliminary prefiguration of the anti-inflammatory capacity of the treatment compounds. Beyond the lowest treatment concentration, the anti-nitrosative activity significantly varied between BRD0705 (GSK3α inhibitor) and BRD3731 (GSK3β inhibitor), which constitutes initial empirical evidence of BRD3731 functional precedency. Comparisons between BRD3731 (GSK3β inhibitor) and BRD0320 (GSK3α/β inhibitor) were not suggestive of a substantial difference in nitrite-reducing capacity due to paralog selectivity, and only at the highest treatment concentrations were differences between BRD0705 (GSK3α inhibitor) and BRD0320 (GSK3α/β inhibitor) determined of statistical import. These results entail that a threshold level of GSK3β inhibition is imperative for a functional drop in nitrite.

To further conceive of the anti-inflammatory potential of paralog-selective GSK3 inhibition, we set out to quantitate the transcriptional rates of several proinflammatory genes using qPCR. Our results demonstrated a significant dose-dependent decrement of the LPS-mediated upregulation of microglial activation markers, which complied with the modulatory trend erstwhile noted, where BRD3731 (GSK3β inhibitor) was the most competent suppressor of LPS-induced proinflammatory upset. Following BRD3731 (GSK3β inhibitor), non-selective GSK3 inhibition by BRD0320 manifested a midrange modulation of these markers, and BRD0705 (GSK3α inhibitor) showed the least, albeit statistically significant reductive potential. The same regulatory pattern was noted with the mRNA expression levels of all the other inflammatory markers tested, namely iNOS, IL-1β, IL-6, and TNF-α. This repressive trend carried through to post-treatment protein levels of the proinflammatory cytokines IL-1β, IL-6, and TNF-α. Our observations agree with earlier work that specifically recognizes GSK3β as the isoform forward-steering inflammatory responses; while maintaining the reliability of regulatory models in which GSK3α is central, such as findings by McAlpine and colleagues, which specify that deletion of GSK3α in myeloid cells promotes an activated “M2” phenotype [51].

We next proceeded with transcript quantitation by qPCR to weigh up the effect of isoform-selective GSK3 inhibition on activating the transcription of the ARE genes, HO-1 and Osgin1. Analysis of all qPCR data indicated that the functional supremacy posed by BRD3731-mediated GSK3β-selective inhibition constituted a statistically robust paradigm. Again, our findings echo earlier reports, such as work by Cuadrado et al., that recognize GSK3β, rather than GSK3α, as a non-canonical negative regulator of Nrf2, as proven by the improved transcriptional activation of the Nrf2-driven ARE genes HO-1 and Osgin1 [23].

According to Wagner, Stegmaier and colleagues, BRD0705, the GSK3α-selective inhibitor, did not affect β-catenin stabilization and was associated with interrupted β-catenin signaling [41]. Given the reported coincidence of β-catenin and Nrf2 deregulation in a number of neurodegenerative contexts [52], documented co-dependency [15], the interplay between the Wnt/β-catenin and NF-κB signaling [53], and another loop involving the LPS-responsive TLR4 [54] — particularly in our investigated pathological context [55, 56] and proposed mechanistic model [52] — we opted to investigate the activity of this transcription factor in response to our treatments. We aimed to verify the variability between the GSK3 isoforms which pose an added advantage in a number of disease groups, in which co-morbidities can be of concern. As such, c-Myc, a target gene of the Wnt/β-catenin signaling [47], was quantitatively assessed by qPCR. GSK3β inhibition was determined to be requisite to nuclear accumulation of β-catenin as can be extrapolated from the marked c-Myc upregulation (as well as the densitometric patterning from immunoblots of β-catenin) following BRD3731 (GSK3β inhibitor) treatment. BRD0320 (GSK3α/β inhibitor) was comparatively delimited and BRD0705 (GSK3α inhibitor) failed to promote any significant increase.

We then looked into whether blunting of GSK3 activity mediates its pro-curative outcomes through tuning the nuclear content of NF-κB and Nrf2. Analysis of nuclear lysates from the relevant treatment groups by Western blotting confirmed that GSK3 inhibitors hindered the nuclear translocation of the p65 subunit of NF-κB and elicited that of Nrf2, echoing the same trend theretofore identified, where BRD3731 (GSK3β inhibitor) displayed the maximum suppressive effect for NF-κB and a notable Nrf2 mobilizing ability. Dual isoform inhibition by BRD0320 considerably tempered the proinflammatory NF-κB nuclear accumulation and enhanced the buildup of the anti-oxidative Nrf2, yet not as effectively as GSK3β-selective inhibition. GSK3α-selective inhibition by BRD0705 was yet again only marginally effective at altering the levels of NF-κB or Nrf2 in the nuclear compartment. Immunoblots were also prepared for β-catenin to determine the extent to which the compounds bear upon Wnt/β-catenin signaling. Interestingly, only samples from BRD3731 (GSK3β inhibitor)-treated cells showed nuclear accumulation of β-catenin; while all other groups consistently failed to signal any discernable nuclear presence of β-catenin. BRD3731 (GSK3β inhibitor) is prominently competent at elevating nuclear β-catenin compared to the other compounds [41], the levels of the β-catenin protein were high enough to show up on our blots, whereas the levels from other samples were too scarce to get picked up.

We finally aimed to question the mechanistic dependency of the anti-inflammatory effects of GSK3 inhibition on Nrf2/ARE signaling. To that end, we sought to silence Nrf2 mRNAs using a pool of 3 different Nrf2-targeting DsiRNAs. None of the GSK3 inhibitors or SFN could upregulate the ARE genes in any considerable manner, which signifies that our knockdown carried through to the protein level and resulted in a transcriptional limitation of its target genes. Finally, the mRNA expression of iNOS, IL-1β, IL-6, and TNF-α was once again determined by qPCR to assess the effect of Nrf2 knockdown on the anti-inflammatory activity of the GSK3 inhibitors as conveyed by the downregulation of these markers. The knockdown of Nrf2 did not affect the anti-inflammatory effects of the GSK3 inhibitors indicating that Nrf2 is non-essential to the anti-inflammatory action of the GSK3 inhibitors.

Collectively, our findings consistently showed that BRD3731 (GSK3β inhibitor) maintained anti-inflammatory and anti-oxidative prepotency over BRD0320 (GSK3α/β inhibitor) and BRD0705 (GSK3α inhibitor), the latter being mildly effective at best. From this steady modulatory pattern, we can derive that GSK3β rather than GSK3α primarily drives inflammatory and pro-oxidative processes, and its inhibition is thus more therapeutically consequential. As such, treatments selectively targeting GSK3β (BRD3731) are likely to be of superior medicinal outcome than same-dose paralog-non-discriminating treatments (BRD0320), where GSK3β is inhibited at a rate that is only fractional of that achieved by targeted inhibition, at any given concentration. Where the dosing regimen is unchanged, treatments selectively targeting GSK3α, with no antagonism of GSK3β whatsoever, produce much milder effects, as proven by our results for BRD0705 (GSK3α inhibitor).

**Fig. 12:**
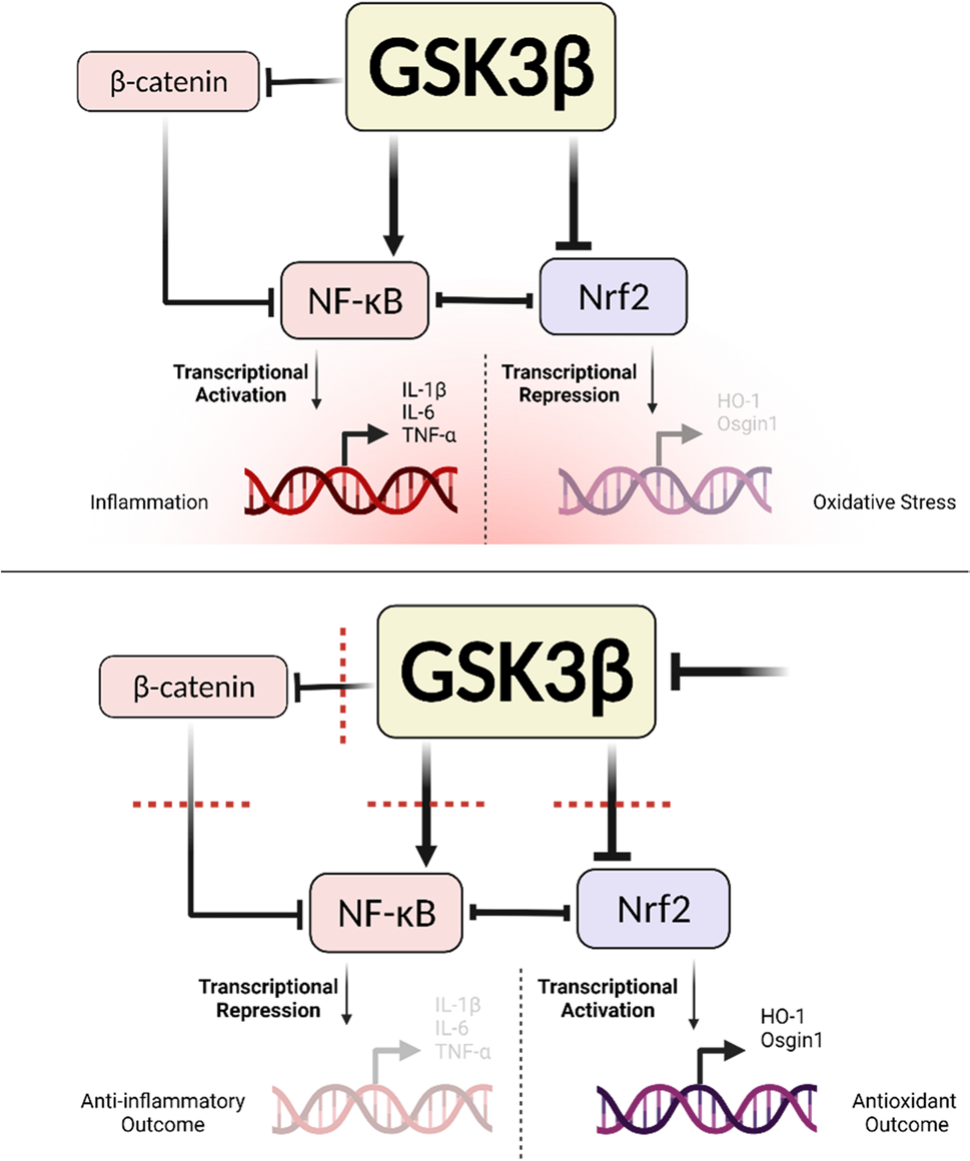
A schematic outlining the GSK3/Nrf2/NF-κB regulatory model underlying GSK3 inhibition in SIM-A9 microglia, as evinced by findings in this study. Created with BioRender.com

Notwithstanding the above, and in light of promising reports that recognize a potential remedial benefit for GSK3α in neurodegenerative/neuroinflammatory pathologies [57–62], targeted inhibition of GSK3α should not be altogether disregarded as a therapeutic strategy, especially in disease groups of known vulnerability to β-catenin-driven pathologies, such as cancer. As evidenced by our experiments, GSK3α inhibition — despite the mediocrity of its effects when compared to GSK3β inhibition –— was capable of moderating pro-pathological molecular changes. On the other hand, the superb selectivity of the BRD0705 for GSK3α combined with its inability to activate β-catenin-mediated transcription unveiled a crucial component in the mechanistic machinery under study. Consistent with the literature [63, 64] and as outlined in Fig. 12, β-catenin seems to be a more consequential player in this loop than was anticipated, whereby its maximal activation by BRD03731 and absence thereof with BRD0705 coincided with attenuated NF-κB signaling and modulation of the inflammatory response in consequence. Such an observation can be corroborated in BRD3731-treated β-catenin knockouts and constitutes a significant future outcome of this work.

Furthermore, and in keeping with previously acknowledged mechanistic models, it would seem that GSK3 exerts its proinflammatory function directly through stimulating NF-κB signaling [65] and promoting the production of proinflammatory molecules (NO, IL-1β, IL-6, and TNF-α), rather than via Nrf2/ARE signaling. The upregulated inflammatory mediators then go on to stimulate the upregulation of the microglial surface markers of activation [66, 67]. It is therefore plausible that while GSK3 is a mutual modulatory factor linking NF-κB activation and Nrf2 inhibition, its regulatory effect on the two pathways is not codependent.

## Conclusion and Future Perspectives

Our data highlights the significance of the dysregulated GSK3/Nrf2/NF-kB regulatory network in activated microglial cells and emphasizes the multimodal therapeutic potential of GSK3 inhibition in neurodegenerative diseases. The evidence herein provided suggests that suppression of GSK3 in activated SIM-A9 cells imparts anti-inflammatory and anti-oxidative effects. Moreover, we present proof that paralog-selectivity of GSK3 inhibition is of the utmost consequence to its anti-inflammatory and anti-oxidative effects, which were maximal with GSK3β-selective inhibition. This signifies the functional superiority of GSK3β to GSK3α, at least as far as its anti-inflammatory and anti-oxidative effects are concerned.

Further examination of paralog-distinctive features within the presented regulatory network in more complex experimental constructs — such as those involving neural organoids and transgenic animal models — would be particularly beneficial since it affords the phenotypic heterogeneity of microglia and can better recapitulate the relevant molecular pathophysiological backdrop. Moreover, the results of this study warrant further investigation of the utility of GSK3α-selective inhibition, especially in experimental set-ups that best recapitulate the pathophysiological contexts and disease groups that can benefit from non-GSK3β-targeting treatments.

Working beyond the limitations of simplistic in vitro systems would provide invaluable insights into the intricate crosstalk between neural subpopulations and offer a more categorical assessment of the molecular mechanisms that are most clinically relevant, which should render the findings of a more translational value.

## Data Availability

The raw data supporting the conclusions of this article will be made available by the authors, without undue reservation.

## Abbreviations

AD: Alzheimer’s disease
ALS: Amyotrophic lateral sclerosis
ARE: Antioxidant response element
CNS: Central nervous system
GSK3: Glycogen synthase kinase 3
HO-1: Heme oxygenase 1
IL-1β: Interleukin-1 beta
IL-6: Interleukin-6
iNOS: Inducible nitric oxide synthase
LPS: Lipopolysaccharide
NDs: Neurodegenerative diseases
Neh: Nrf2-ECH homology modules
NF-κB: Nuclear factor kappa-light-chain-enhancer of activated B cells
NO: Nitric oxide
Nrf2: Nuclear factor erythroid 2-related factor 2
Osgin1: Oxidative stress induced growth inhibitor 1
PD: Parkinson’s disease
P-tau: Hyperphosphorylated tau
SIM-A9: Spontaneously immortalized microglia, clone A9
TLR: Toll-like receptor
TNF-α: Tumor necrosis factor-α

## References

[1] DiSabato D, Quan N, Godbout JP (2016) Neuroinflammation: the devil is in the details. J Neurochem [Internet]. [cited 2021 Sep 8];139(Suppl 2):136. Available from: https://www.ncbi.nlm.nih.gov/pmc/articles/PMC5025335/10.1111/jnc.13607 PMC5025335 26990767

[2] Chitnis T, Weiner HL. CNS inflammation and neurodegeneration. J Clin Invest [Internet]. 2017 Oct 2 [cited 2021 Sep 8];127(10):3577–87. Available from: 10.1172/JCI90609 10.1172/JCI90609 PMC5617655 28872464

[3] Guzman-Martinez L, Maccioni RB, Andrade V, Navarrete LP, Pastor MG, Ramos-Escobar N (2019) Neuroinflammation as a Common Feature of Neurodegenerative Disorders. Front Pharmacol 10:1008. 10.3389/fphar.2019.01008 10.3389/fphar.2019.01008 PMC6751310 31572186

[4] Kwon HS, Koh S-H. Neuroinflammation in neurodegenerative disorders: the roles of microglia and astrocytes. Transl Neurodegener 2020 91 [Internet]. 2020 Nov 26 [cited 2021 Sep 8];9(1):1–12. Available from: https://translationalneurodegeneration.biomedcentral.com/articles/10.1186/s40035-020-00221-2 10.1186/s40035-020-00221-2 PMC7689983 33239064

[5] Ransohoff RM (2016) How neuroinflammation contributes to neurodegeneration. Science (80- ) 353(6301):777–83 10.1126/science.aag2590 27540165

[6] Graeber MB, Li W, Rodriguez ML (2011 Dec 1) Role of microglia in CNS inflammation. FEBS Lett. 585(23):3798–805. 10.1016/j.febslet.2011.08.033 10.1016/j.febslet.2011.08.033 21889505

[7] Streit WJ, Mrak RE, Griffin WST (2004) Microglia and neuroinflammation: a pathological perspective. J Neuroinflammation [Internet]. [cited 2021 Mar 26];1(1):14. Available from: http://jneuroinflammation.biomedcentral.com/articles/10.1186/1742-2094-1-14 10.1186/1742-2094-1-14 PMC509427 15285801

[8] Streit WJ. Microglia as neuroprotective, immunocompetent cells of the CNS. Glia [Internet]. 2002 Nov 1 [cited 2021 Oct 11];40(2):133–9. Available from: https://onlinelibrary.wiley.com/doi/full/10.1002/glia.10154 10.1002/glia.10154 12379901

[9] Leyh J, Paeschke S, Mages B, Michalski D, Nowicki M, Bechmann I (2021). Classification of microglial morphological phenotypes using machine learning. Front Cell Neurosci.

[10] Hemonnot A-L, Hua J, Ulmann L, Hirbec H (2019) Microglia in Alzheimer Disease: Well-Known Targets and New Opportunities. Front Aging Neurosci 0(JUL):233 10.3389/fnagi.2019.00233 PMC6730262 31543810

[11] Stelzmann RA, Schnitzlein HN, Murtagh FR. An english translation of alzheimer’s 1907 paper, “über eine eigenartige erkankung der hirnrinde.” Clin Anat [Internet]. 1995 Jan 1 [cited 2021 Oct 20];8(6):429–31. Available from: https://onlinelibrary.wiley.com/doi/full/10.1002/ca.980080612 10.1002/ca.980080612 8713166

[12] Graeber MB, Kösel S, Egensperger R, Banati RB, Müller U, Bise K et al. Rediscovery of the case described by Alois Alzheimer in 1911: historical, histological and molecular genetic analysis. Neurogenetics 1997 11 [Internet]. 1997 [cited 2021 Oct 20];1(1):73–80. Available from: https://link.springer.com/article/10.1007/s100480050011 10.1007/s100480050011 10735278

[13] Llano D, Li J, Waring J, Ellis T, Devanarayan V, Witte D et al. Cerebrospinal fluid cytokine dynamics differ between Alzheimer disease patients and elderly controls. Alzheimer Dis Assoc Disord [Internet]. 2012 Oct [cited 2021 Oct 20];26(4):322–8. Available from: https://pubmed.ncbi.nlm.nih.gov/22089638/10.1097/WAD.0b013e31823b2728 22089638

[14] Chen X, Hu Y, Cao Z, Liu Q, Cheng Y (2018) Cerebrospinal fluid inflammatory cytokine aberrations in Alzheimer’s disease, Parkinson’s disease and amyotrophic lateral sclerosis: a systematic review and meta-analysis. Front Immunol [Internet]. [cited 2021 Oct 20];9(SEP). Available from: https://www.ncbi.nlm.nih.gov/pmc/articles/PMC6156158/10.3389/fimmu.2018.02122 PMC6156158 30283455

[15] Marchetti B. Nrf2/Wnt resilience orchestrates rejuvenation of glia-neuron dialogue in Parkinson’s disease. Redox Biol [Internet]. 2020 Aug 1 [cited 2021 Sep 15];36:101664–101664. Available from: https://europepmc.org/articles/PMC7395594 10.1016/j.redox.2020.101664 PMC7395594 32863224

[16] L’Episcopo F, Tirolo C, Testa N, Caniglia S, Morale M, Deleidi M et al. Plasticity of subventricular zone neuroprogenitors in MPTP (1-methyl-4-phenyl-1,2,3,6-tetrahydropyridine) mouse model of Parkinson’s disease involves cross talk between inflammatory and Wnt/β-catenin signaling pathways: functional consequences for neuropr. J Neurosci [Internet]. 2012 Feb 1 [cited 2021 Sep 15];32(6):2062–85. Available from: https://www.ncbi.nlm.nih.gov/pmc/articles/pmid/22323720/?tool=EBI 10.1523/JNEUROSCI.5259-11.2012 PMC3556384 22323720

[17] L’episcopo F, Serapide M, Tirolo C, Testa N, Caniglia S, Morale M et al. A Wnt1 regulated Frizzled-1/β-Catenin signaling pathway as a candidate regulatory circuit controlling mesencephalic dopaminergic neuron-astrocyte crosstalk: Therapeutical relevance for neuron survival and neuroprotection. Mol Neurodegener [Internet]. 2011 Jul 13 [cited 2021 Sep 15];6(1):49–49. Available from: https://www.ncbi.nlm.nih.gov/pmc/articles/pmid/21752258/?tool=EBI 10.1186/1750-1326-6-49 PMC3162575 21752258

[18] L’Episcopo F, Tirolo C, Testa N, Caniglia S, Morale M, Impagnatiello F et al. Aging-induced Nrf2-ARE pathway disruption in the subventricular zone drives neurogenic impairment in parkinsonian mice via PI3K-Wnt/β-catenin dysregulation. J Neurosci [Internet]. 2013 Jan 1 [cited 2021 Sep 15];33(4):1462–85. Available from: https://www.ncbi.nlm.nih.gov/pmc/articles/pmid/23345222/?tool=EBI 10.1523/JNEUROSCI.3206-12.2013 PMC3564519 23345222

[19] L’Episcopo F, Tirolo C, Testa N, Caniglia S, Morale M, Serapide M et al. Wnt/β-catenin signaling is required to rescue midbrain dopaminergic progenitors and promote neurorepair in ageing mouse model of Parkinson’s disease. Stem Cells [Internet]. 2014 Aug 1 [cited 2021 Sep 15];32(8):2147–63. Available from: https://www.ncbi.nlm.nih.gov/pmc/articles/pmid/24648001/?tool=EBI 10.1002/stem.1708 PMC4106883 24648001

[20] Marchetti B, Pluchino S. Wnt your brain be inflamed? Yes, it Wnt! Trends Mol Med [Internet]. 2013 Jan 9 [cited 2021 Sep 15];19(3):144–56. Available from: https://www.ncbi.nlm.nih.gov/pmc/articles/pmid/23312954/?tool=EBI 10.1016/j.molmed.2012.12.001 PMC3595301 23312954

[21] Harvey K, Marchetti B. Regulating Wnt signaling: a strategy to prevent neurodegeneration and induce regeneration. J Mol Cell Biol [Internet]. 2014 Feb 1 [cited 2021 Sep 15];6(1):1–2. Available from: https://europepmc.org/article/MED/24549156 10.1093/jmcb/mju002 24549156

[22] L’Episcopo F, Tirolo C, Testa N, Caniglia S, Morale M, Cossetti C et al. Reactive astrocytes and Wnt/β-catenin signaling link nigrostriatal injury to repair in 1-methyl-4-phenyl-1,2,3,6-tetrahydropyridine model of Parkinson’s disease. Neurobiol Dis [Internet]. 2010 Nov 5 [cited 2021 Sep 15];41(2):508–27. Available from: https://www.ncbi.nlm.nih.gov/pmc/articles/pmid/21056667/?tool=EBI 10.1016/j.nbd.2010.10.023 PMC3558878 21056667

[23] Cuadrado A, Kügler S, Lastres-Becker I (2018) Pharmacological targeting of GSK-3 and NRF2 provides neuroprotection in a preclinical model of tauopathy. Redox Biol [Internet]. [cited 2021 Sep 12];14:522–34. Available from: https://www.ncbi.nlm.nih.gov/pmc/articles/PMC5681345/10.1016/j.redox.2017.10.010 PMC5681345 29121589

[24] Rada P, Rojo AI, Chowdhry S, McMahon M, Hayes JD, Cuadrado A. SCF/-TrCP promotes glycogen synthase kinase 3-dependent degradation of the Nrf2 transcription factor in a Keap1-independent manner. Mol Cell Biol [Internet]. 2011 Mar 15 [cited 2021 Sep 20];31(6):1121–33. Available from: https://journals.asm.org/journal/mcb 10.1128/MCB.01204-10 PMC3067901 21245377

[25] Yamazaki H, Tanji K, Wakabayashi K, Matsuura S, Itoh K. Role of the Keap1/Nrf2 pathway in neurodegenerative diseases. Pathol Int [Internet]. 2015 May 1 [cited 2021 Sep 15];65(5):210–9. Available from: https://onlinelibrary.wiley.com/doi/full/10.1111/pin.12261 10.1111/pin.12261 25707882

[26] Kumar H, Lim HW, More SV, Kim BW, Koppula S, Kim IS et al. The role of free radicals in the aging brain and Parkinson’s disease: convergence and parallelism. Int J Mol Sci [Internet]. 2012 [cited 2021 Sep 15];13(8):10478–504. Available from: https://pubmed.ncbi.nlm.nih.gov/22949875/10.3390/ijms130810478 PMC3431873 22949875

[27] Medunjanin S, Schleithoff L, Fiegehenn C, Weinert S, Zuschratter W, Braun-Dullaeus RC. GSK-3β controls NF-kappaB activity via IKKγ/NEMO. Sci Reports 2016 61 [Internet]. 2016 Dec 8 [cited 2021 Sep 21];6(1):1–11. Available from: https://www.nature.com/articles/srep38553 10.1038/srep38553 PMC5144080 27929056

[28] Hoffmeister L, Diekmann M, Brand K, Huber R (2020) GSK3: A kinase balancing promotion and resolution of inflammation. Cells [Internet]. [cited 2021 Sep 11];9(4). Available from: https://www.ncbi.nlm.nih.gov/pmc/articles/PMC7226814/10.3390/cells9040820 PMC7226814 32231133

[29] Lee G, Leugers CJ (2012) Tau and tauopathies. Prog Mol Biol Transl Sci [Internet]. [cited 2021 Sep 12];107:263. Available from: https://www.ncbi.nlm.nih.gov/pmc/articles/PMC3614411/10.1016/B978-0-12-385883-2.00004-7 PMC3614411 22482453

[30] Avila J, León-Espinosa G, García E, García-Escudero V, Hernández F, Defelipe J (2012) Tau Phosphorylation by GSK3 in Different Conditions. International journal of Alzheimer’s disease. 10.1155/2012/578373 10.1155/2012/578373 PMC3362846 22675648

[31] Hanger DP, Anderton BH, Noble W. Tau phosphorylation: the therapeutic challenge for neurodegenerative disease. Trends Mol Med [Internet]. 2009 Mar 1 [cited 2021 Sep 12];15(3):112–9. Available from: http://www.cell.com/article/S1471491409000331/fulltext 10.1016/j.molmed.2009.01.003 19246243

[32] Ramkumar A, Jong BY, Ori-McKenney KM. ReMAPping the microtubule landscape: How phosphorylation dictates the activities of microtubule-associated proteins. Dev Dyn [Internet]. 2018 Jan 1 [cited 2021 Sep 12];247(1):138–55. Available from: https://onlinelibrary.wiley.com/doi/full/10.1002/dvdy.24599 10.1002/dvdy.24599 PMC5739964 28980356

[33] Ajmone-Cat MA, D’Urso MC, di Blasio G, Brignone MS, De Simone R, Minghetti L (2016). Glycogen synthase kinase 3 is part of the molecular machinery regulating the adaptive response to LPS stimulation in microglial cells. Brain Behav Immun.

[34] Yuskaitis CJ, Jope RS, CJ Y, RS J, Yuskaitis CJ, Jope RS. Glycogen synthase kinase-3 regulates microglial migration, inflammation, and inflammation-induced neurotoxicity. Cell Signal [Internet]. 2009 Feb [cited 2021 Sep 11];21(2). Available from: https://pubmed.ncbi.nlm.nih.gov/19007880/10.1016/j.cellsig.2008.10.014 PMC2630396 19007880

[35] Beurel E, Jope RS, E B, RS J, Beurel E, Jope RS et al. Lipopolysaccharide-induced interleukin-6 production is controlled by glycogen synthase kinase-3 and STAT3 in the brain. J Neuroinflammation 2009 61 [Internet]. 2009 Mar 11 [cited 2021 Sep 11];6(1):1–11. Available from: https://pubmed.ncbi.nlm.nih.gov/19284588/10.1186/1742-2094-6-9 PMC2660311 19284588

[36] Cheng Y, Wang C, Huang W, Tsai C, Chen C, Shen C et al. Staphylococcus aureus induces microglial inflammation via a glycogen synthase kinase 3beta-regulated pathway. Infect Immun [Internet]. 2009 Sep [cited 2021 Sep 11];77(9):4002–8. Available from: https://pubmed.ncbi.nlm.nih.gov/19596777/10.1128/IAI.00176-09 PMC2738015 19596777

[37] Li D, Liu Z, Chen W, Yao M, Li G. Association of glycogen synthase kinase‑3β with Parkinson’s disease (Review). Mol Med Rep [Internet]. 2014 Jun 1 [cited 2021 Sep 11];9(6):2043–50. Available from: http://www.spandidos-publications.com/10.3892/mmr.2014.2080/abstract 10.3892/mmr.2014.2080 PMC4055480 24681994

[38] Woodgett JR (1991). cDNA cloning and properties of glycogen synthase kinase-3. Methods Enzymol.

[39] Terwel D, Muyllaert D, Dewachter I, Borghgraef P, Croes S, Devijver H et al (2008) Amyloid activates GSK-3β to aggravate neuronal tauopathy in bigenic mice. Am J Pathol [Internet]. [cited 2021 Sep 11];172(3):786. Available from: https://www.ncbi.nlm.nih.gov/pmc/articles/PMC2258274/10.2353/ajpath.2008.070904 PMC2258274 18258852

[40] Sutherland C (2011) What are the bona fide GSK3 substrates? Int J Alzheimers Dis [Internet]. [cited 2021 Sep 11];2011:24. Available from: https://www.ncbi.nlm.nih.gov/pmc/articles/PMC3100594/10.4061/2011/505607 PMC3100594 21629754

[41] Wagner FF, Benajiba L, Campbell AJ, Weïwer M, Sacher JR, Gale JP, Ross L, Puissant A, Alexe G, Conway A, Back M, Pikman Y, Galinsky I, DeAngelo DJ, Stone RM, Kaya T, Shi X, Robers MB, Machleidt T, Wilkinson J, … Holson EB (2018) Exploiting an Asp-Glu "switch" in glycogen synthase kinase 3 to design paralog-selective inhibitors for use in acute myeloid leukemia. Science Translational Medicine 10(431). 10.1126/scitranslmed.aam8460 10.1126/scitranslmed.aam8460 PMC6553635 29515000

[42] Ladeby R, Wirenfeldt M, Garcia-Ovejero D, Fenger C, Dissing-Olesen L, Dalmau I (2005). Microglial cell population dynamics in the injured adult central nervous system. Brain Res Rev.

[43] Ohsawa K, Imai Y, Sasaki Y, Kohsaka S. Microglia/macrophage-specific protein Iba1 binds to fimbrin and enhances its actin-bundling activity. J Neurochem [Internet]. 2004 Feb 1 [cited 2021 Oct 4];88(4):844–56. Available from: https://onlinelibrary.wiley.com/doi/full/10.1046/j.1471-4159.2003.02213.x 10.1046/j.1471-4159.2003.02213.x 14756805

[44] Xue Q, Yan Y, Zhang R, Xiong H (2018) Regulation of iNOS on immune cells and its role in diseases. Int J Mol Sci [Internet]. [cited 2021 Oct 4];19(12). Available from: https://www.ncbi.nlm.nih.gov/pmc/articles/PMC6320759/10.3390/ijms19123805 PMC6320759 30501075

[45] Suschek C, Schnorr O, Kolb-Bachofen V. The role of iNOS in chronic inflammatory processes in vivo: is it damage-promoting, protective, or active at all? Curr Mol Med [Internet]. 2004 Mar 18 [cited 2021 Oct 4];4(7):763–75. Available from: https://pubmed.ncbi.nlm.nih.gov/15579023/10.2174/1566524043359908 15579023

[46] Wang W-Y, Tan M-S, Yu J-T, Tan L. Role of pro-inflammatory cytokines released from microglia in Alzheimer’s disease. Ann Transl Med [Internet]. 2015 Jun 1 [cited 2021 Oct 5];3(10):7–7. Available from: https://atm.amegroups.com/article/view/6546/7583 10.3978/j.issn.2305-5839.2015.03.49 PMC4486922 26207229

[47] Herbst A, Jurinovic V, Krebs S, Thieme SE, Blum H, Göke B, et al. Comprehensive analysis of β-catenin target genes in colorectal carcinoma cell lines with deregulated Wnt/β-catenin signaling. BMC Genomics 2014 151 [Internet]. 2014 Jan 28 [cited 2021 Oct 5];15(1):1–15. Available from: https://bmcgenomics.biomedcentral.com/articles/10.1186/1471-2164-15-74 10.1186/1471-2164-15-74 PMC3909937 24467841

[48] Nagamoto-Combs K, Kulas J, Combs CK (2014) A novel cell line from spontaneously immortalized murine microglia. J Neurosci Methods [Internet]. [cited 2021 Jun 12];233:187–98. Available from: https://pubmed.ncbi.nlm.nih.gov/24975292/10.1016/j.jneumeth.2014.05.021 PMC4140094 24975292

[49] Dave KM, Ali L, Manickam DS. Characterization of the SIM-A9 cell line as a model of activated microglia in the context of neuropathic pain. PLoS One [Internet]. 2020 Apr 1 [cited 2021 Jun 12];15(4):e0231597. Available from: 10.1371/journal.pone.0231597 10.1371/journal.pone.0231597 PMC7156095 32287325

[50] Desforges NM, Hebron ML, Algarzae NK, Lonskaya I, Moussa CEH (2012) Fractalkine mediates communication between pathogenic proteins and microglia: Implications of anti-inflammatory treatments in different stages of neurodegenerative diseases. Int J Alzheimers Dis 10.1155/2012/345472 PMC3420133 22919540

[51] Mcalpine CS, Huang A, Emdin A, Banko NS, Beriault DR, Shi Y et al. Deletion of myeloid GSK3α attenuates atherosclerosis and promotes an M2 macrophage phenotype. Arterioscler Thromb Vasc Biol [Internet]. 2015 May 27 [cited 2022 Jan 12];35(5):1113–22. Available from: https://www.ahajournals.org/doi/abs/10.1161/ATVBAHA.115.305438 10.1161/ATVBAHA.115.305438 25767272

[52] Gendy A, Soubh A, Al-Mokaddem A, Kotb E-S (2021). Dimethyl fumarate protects against intestinal ischemia/reperfusion lesion: participation of Nrf2/HO-1, GSK-3β and Wnt/β-catenin pathway. Biomed Pharmacother.

[53] Ma B, Hottiger MO (2016). Crosstalk between Wnt/β-Catenin and NF-κB Signaling Pathway during Inflammation. Frontiers in Immunology.

[54] Zolezzi JM, Inestrosa NC (2017). Wnt/TLR Dialog in Neuroinflammation, Relevance in Alzheimer's Disease. Frontiers in Immunology.

[55] Jia L, Piña-Crespo J, Li Y. Restoring Wnt/β-catenin signaling is a promising therapeutic strategy for Alzheimer’s disease. Mol Brain 2019 121 [Internet]. 2019 Dec 4 [cited 2021 Oct 5];12(1):1–11. Available from: https://molecularbrain.biomedcentral.com/articles/10.1186/s13041-019-0525-5 10.1186/s13041-019-0525-5 PMC6894260 31801553

[56] Orellana AMM, Vasconcelos AR, Leite JA, Lima L de S, Andreotti DZ, Munhoz CD et al (2015) Age-related neuroinflammation and changes in AKT-GSK-3β and WNT/ β-CATENIN signaling in rat hippocampus. Aging (Albany NY) [Internet]. [cited 2021 Oct 5];7(12):1094. Available from: https://www.ncbi.nlm.nih.gov/pmc/articles/PMC4712335/10.18632/aging.100853 PMC4712335 26647069

[57] Draffin JE, Sánchez-Castillo C, Fernández-Rodrigo A, Sánchez-Sáez X, Ávila J, Wagner FF et al. GSK3α, not GSK3β, drives hippocampal NMDAR-dependent LTD via tau-mediated spine anchoring. EMBO J [Internet]. 2021 Jan 15 [cited 2021 Oct 27];40(2):e105513. Available from: https://onlinelibrary.wiley.com/doi/full/10.15252/embj.2020105513 10.15252/embj.2020105513 PMC7809792 33197065

[58] Kaidanovich-Beilin O, Lipina T V, Takao K, van Eede M, Hattori S, Laliberté C et al. Abnormalities in brain structure and behavior in GSK-3alpha mutant mice. Mol Brain 2009 21 [Internet]. 2009 Nov 19 [cited 2021 Oct 27];2(1):1–23. Available from: https://molecularbrain.biomedcentral.com/articles/10.1186/1756-6606-2-35 10.1186/1756-6606-2-35 PMC2785804 19925672

[59] Dunning CJ, McGauran G, Willén K, Gouras GK, O’Connell DJ, Linse S. Direct high affinity interaction between Aβ42 and GSK3α stimulates hyperphosphorylation of tau. A new molecular link in Alzheimer’s disease? ACS Chem Neurosci [Internet]. 2015 Feb 17 [cited 2021 Oct 27];7(2):161–70. Available from: https://pubs.acs.org/doi/full/10.1021/acschemneuro.5b00262 10.1021/acschemneuro.5b00262 PMC4759616 26618561

[60] McAlpine CS, Huang A, Emdin A, Banko NS, Beriault DR, Shi Y et al. Deletion of myeloid GSK3α attenuates atherosclerosis and promotes an M2 macrophage phenotype. Arterioscler Thromb Vasc Biol [Internet]. 2015 May 27 [cited 2021 Oct 27];35(5):1113–22. Available from: https://www.ahajournals.org/doi/abs/10.1161/ATVBAHA.115.305438 10.1161/ATVBAHA.115.305438 25767272

[61] Zhou J, Freeman TA, Ahmad F, Shang X, Mangano E, Gao E et al. GSK-3α is a central regulator of age-related pathologies in mice. J Clin Invest [Internet]. 2013 Apr 1 [cited 2021 Oct 27];123(4):1821. Available from: /pmc/articles/PMC3613907/10.1172/JCI64398 PMC3613907 23549082

[62] Phiel CJ, Wilson CA, Lee VM-Y, Klein PS (2003) GSK-3α regulates production of Alzheimer’s disease amyloid-β peptides. Nat 2003 4236938 [Internet]. [cited 2021 Oct 27];423(6938):435–9. Available from: https://www.nature.com/articles/nature01640 10.1038/nature01640 12761548

[63] Ougolkov A V., Billadeau DD. Targeting GSK-3: A promising approach for cancer therapy? Futur Oncol [Internet]. 2006 Feb 23 [cited 2022 Jan 14];2(1):91–100. Available from: https://www.futuremedicine.com/doi/abs/10.2217/14796694.2.1.91 10.2217/14796694.2.1.91 16556076

[64] Koistinaho J, Malm T, Goldsteins G (2011) Glycogen synthase kinase-3β: a mediator of inflammation in Alzheimer’s disease? Int J Alzheimers Dis 10.4061/2011/129753 PMC3100542 21629736

[65] Hoeflich KP, Luo J, Rubie EA, Tsao MS, Jin O, Woodgett JR (2000) Requirement for glycogen synthase kinase-3beta in cell survival and NF-kappaB activation. Nature 406(6791):86–90. 10.1038/35017574 10.1038/35017574 10894547

[66] Roy A, Fung YK, Liu X, Pahan K. Up-regulation of Microglial CD11b Expression by nitric oxide. J Biol Chem [Internet]. 2006 May 26 [cited 2021 Oct 25];281(21):14971. Available from: /pmc/articles/PMC1963414/10.1074/jbc.M600236200 PMC1963414 16551637

[67] Zhou X, Gao X-P, Fan J, Liu Q, Anwar KN, Frey RS, et al (2005) LPS activation of Toll-like receptor 4 signals CD11b/CD18 expression in neutrophils. 10.1152/ajplung003272004 [Internet]. [cited 2021 Oct 25] 288(4 32–4):655–62. Available from: 10.1152/ajplung.00327.2004

